# TRPV2 regulates cell fate in the human granulosa-like tumor cell line KGN: implications for granulosa cell tumors and cannabidiol

**DOI:** 10.1186/s12964-026-02729-y

**Published:** 2026-02-12

**Authors:** Katja Eubler, Sree Priyanka Jeevanandan, Karolina Magdalena Caban, Jan Bernd Stöckl, Malte Benjamin Braun, Carola Herrmann, Michaela Schneider, Nicole Kreitmair, Lina Scholz, Doris Mayr, Jörg Renkawitz, Harald Welter, Annette Müller-Taubenberger, Thomas Fröhlich, Artur Mayerhofer

**Affiliations:** 1 Biomedical Center Munich (BMC), Cell Biology, Anatomy III, Faculty of Medicine, Ludwig Maximilian University (LMU) Munich, Planegg-Martinsried, Germany; 2 Laboratory for Functional Genome Analysis LAFUGA, Gene Center, LMU Munich, Munich, Germany; 3 Biomedical Center and Walter Brendel Center of Experimental Medicine, Institute of Cardiovascular Physiology and Pathophysiology, University Hospital, LMU Munich, Munich, Germany; 4 Institute of Pathology, University Hospital, LMU Munich, Munich, Germany

**Keywords:** Ovary, Granulosa cell tumor, Cell death, Biomarker, Drug target

## Abstract

**Background:**

The transient receptor potential vanilloid 2 (TRPV2) channel is known to have strong species-dependent activation modes and divers functions. Based on previous results, which showed expression by human ovarian granulosa cells, *w*e studied human TRPV2 in human granulosa cell tumors (GCTs) and derived cells (KGN cells). GCTs are rare ovarian tumors, for which neither specific therapies, nor adequate markers are available.

**Methods:**

We analyzed primary GCTs, including a panel of 63 GCT samples, and KGN cells. We performed immunohisto-/cytochemistry, RT-PCR, Western blotting and measurements of intracellular Ca^2+^ levels. We studied consequences of CRISPR/Cas9-based deletion of TRPV2 on cell proliferation, migration and macropinocytotic behavior, examined changes in steroid hormone production, and determined corresponding alterations of the proteome of these cells.

**Results:**

We found that GCTs express TRPV2 to a large percentage (95%). To examine roles of TRPV2, we turned to the human GCT-derived KGN cell line. CRISPR/Cas9-based deletion of TRPV2 resulted in larger cell size, increases in proliferation, migration and macropinocytotic behavior, changes in steroid production, and corresponding alterations of the proteome of these cells. Deletion of TRPV2 also significantly reduced susceptibility to cell death, which was induced within hours by cannabidiol (CBD), a preferred ligand of TRPV2, in a concentration- and time-dependent manner. However, cell death was not completely abolished and the analysis of the TRPV2-interactome suggested the voltage-dependent anion channel 1 (VDAC1) as a further target for CBD. VDAC1, as part of a cascade involving formation and persistent opening of the mitochondrial permeability transition pore (mPTP), is linked to cell death. A blocker of mPTP formation, cyclosporin A, significantly decreased the vulnerability of KGN cells to CBD-induced cell death. TRPV2-depleted KGN cells treated with cyclosporin A became almost completely insensitive to the effects of CBD.

**Conclusions:**

The results reveal a role of TRPV2 in GCT cells. Thus, CBD causes cell death in KGN cells via direct TRPV2 activation and via interaction with VDAC1. Expression of TRPV2 may thus be a novel marker to distinguish subtypes of GCTs. Furthermore, TRPV2 represents a novel drug target.

**Supplementary Information:**

The online version contains supplementary material available at 10.1186/s12964-026-02729-y.

## Introduction

 The superfamily of transient receptor potential (TRP) channels comprises a total of 28 ubiquitously expressed members in mammals [1]. They are involved in physiological and pathophysiological processes. Some of these channels are well-examined, such as TRP vanilloid 1 (TRPV1) [2, 3], while others are not, including TRPV2.

As a nonselective cation channel, TRPV2 was originally described as translocating calcium channel functioning as a thermosensor [4, 5]. However, knockout mice did not show impaired thermal nociception, but were susceptible to perinatal lethality [6]. Also, noxious heat > 50° is only able to activate rodent, but not human TRPV2 [7], arguing for species-dependent activation modes and functions. TRPV2, in general, has been described to be involved in several physiological processes, including neuronal outgrowth, mechano-sensation, myocardial function, innate and adaptive immunity, endocrine secretion and endometrial development [8–11]. Furthermore, TRPV2 has been associated with invasiveness and growth of many tumor types [10, 12–14] and was suggested to have oncogenic potential (including breast cancer, prostate cancer, others). In contrast, TRPV2 was reduced in high-grade gliomas, results that may indicate a negative role in tumor progression. In support, silencing of TRPV2 in a glioma cell line (U87MG) resulted in the modulation of genes promoting cell proliferation and survival and thereby increased cell survival and proliferation [13].

We previously identified TRPV2 in cells of the human ovary [15], including human granulosa cells (hGCs). In primary hGCs, derived from in vitro fertilization patients, channel activation by the activator cannabidiol (CBD) [16–18] acutely increased levels of intracellular Ca^2+^ and stimulated the secretion of inflammatory factors, including interleukin (IL) 6 and 8 [15]. TRPV2 may therefore be involved in the regulation of inflammatory processes and thereby may contribute to ovarian (patho)physiology.

Granulosa cell tumors (GCTs) are rare tumors, which are derived from follicular hGCs. They cause 2–5% of all ovarian cancers and are potentially malignant stromal tumor types. The majority of GCTs are adult-type GCTs, which bear a missense mutation of forkhead box L2 (FOXL2). Typically, treatment includes the surgical removal of the tumor tissue followed by chemotherapy, but specific treatment options are limited [19, 20]. Furthermore, adequate prognostic markers are not well known yet [21].

A widely used cellular model for the study of adult-type GCTs is the KGN cell line, which originated from a 63-year-old woman. These cells exhibit the same steroidogenic pattern as normal hGCs and bear the FOXL2 mutation [22–24]. Based on a previous study, KGN cells and many primary GCTs express the Ca^2+^ permeable transient receptor potential melastatin 2 (TRPM2), another member of the TRP channel family [25]. We found that H_2_O_2_ activates TRPM2 and promotes susceptibility to cell death in KGN cells. We concluded that induction of oxidative stress may be beneficial in GCT therapy and that TRPM2 may serve as a drug target in GCTs.

However, the role for TRPV2 in these GCTs has not been investigated. Because pilot data indicated expression of TRPV2 in three GCT samples and in KGN cells, the initial purpose of this study was to fill this gap of knowledge. To explore roles of TRPV2, we studied consequences of TRPV2 activation and examined consequences of TRPV2-deletion, employing the CRISPR/Cas9 technique. The results reveal a fundamental role of TRPV2 in shaping cell fate and the regulation of cell death of KGN cells. As TRPV2 is expressed in variable degrees by human GCTs, TRPV2 may be a novel marker to distinguish subtypes of GCTs. Furthermore, targeting TRPV2 could be beneficial in the treatment of GCTs.

## Materials & methods

### Human granulosa cell tumors & tissue microarrays

The expression of TRPV2 in human granulosa cell tumors (GCTs) was evaluated using mRNA extracted from post-operative tumor samples derived from three patients (53 ± 7 years of age), diagnosed at the Institute of Pathology at Ludwig Maximilian University (LMU) of Munich. These samples were described in a previous report [26]. All patients agreed to the scientific use of the tissue (informed consent) and the study was approved by the ethical committee of LMU (project 390 − 15). In addition, archival material of tissue microarrays (TMAs) consisting of 63 GCT samples was used for immunohistochemistry. Information about these samples and the patients is provided in Supplementary Fig. 1. This part of the study was also approved by the ethical committee of LMU (projects 20–697 & 20–895). All experiments were performed in accordance with the relevant guidelines and regulations.

### Immunohistochemistry & immunocytochemistry

The TMAs were subjected to immunohistochemistry and KGN cells seeded on glass coverslips (2-2.5 × 10^4^ cells/coverslip) were used for immunocytochemistry, as described earlier [26, 27]. For both methods, primary polyclonal IgG rabbit antibodies targeted against TRPV2 (HPA044993, 1:400; Atlas Antibodies, Stockholm, Sweden) or VDAC1 (10866-1-AP, 1:1,000; Proteintech, Rosemont, IL, USA) were used, and omission of the primary antibody, rabbit IgG controls, and in case of TRPV2 also pre-adsorption with the adequate blocking peptide (APREST83822, Sigma-Aldrich, St. Louis, MI, USA) served as negative controls, as reported previously [15]. For double labeling studies we used an antibody recognizing the endoplasmic reticulum marker calreticulin (directly Alexa Fluor 488-conjugated; #62304S, 1:200; Cell Signaling, Danvers, MA, USA) in combination with the TRPV2 antibody. Furthermore, for double-labelling of TRPV2 and VDAC, we used the TRPV2 antibody in combination with a mouse anti-VDAC1/Porin + VDAC3 antibody [20B12AF2; 1:200; abcam (Cambridge; UK; Supplementary Fig. 2).

### KGN cells & culture conditions

The human ovarian granulosa-like tumor cell line KGN was obtained from the RIKEN BioResource Research Centre in Ibaraki, Japan [22]. KGN cells were cultured in Dulbecco’s Modified Eagle Medium/Nutrient Mixture F-12 (DMEM/F-12; Thermo Fisher Scientific, Waltham, MA, USA) supplemented with 10% fetal calf serum (FCS; Capricorn Scientific, Ebsdorfergrund, Germany) and 1% penicillin/streptomycin (P/S; Biochrom, Berlin, Germany) and were kept under standard cell culture conditions (37 °C, 5% CO_2_, 95% humidity), as described earlier [28]. Cell passage numbers for the experiments described ranged between 25 and 45.

### CRISPR/Cas9 gene editing

TRPV2-deficient KGN clones were generated using the CRISPR/Cas9 technique. To this end, KGN cells were transfected for 24 h with a PX459 plasmid vector (pSpCas9(BB)-2A-Puro, #62988; addgene, Watertown, MA, USA) mixed with FuGeneHD transfection reagent (Promega, Madison, WI, USA). This plasmid contained, alongside the Cas9 gene and a cassette for puromycin resistance (PuroR), selected guide RNAs targeting exon 12 of the TRPV2 coding region (chromosome 17, p11.2; 5’-3’: 2165–2184 & 2301–3221; designed with e-crisp.org) leading to a deletion of 122 bp (5‘… CATCGGCATGGGCGAGCTGGCCTTCCAGGAGCAGCTGCACTTCCGCGGCATGGTGCTGCTGCTGCTGCTGGCCTACGTGCTGCTCACCTACATCCTGCTGCTCAACATGCTCATCGCCCTCA…3‘; more detailed information, see Supplementary Fig. 3). After transfection, cells were kept in culture medium supplemented with 0.5 µg/ml puromycin (Thermo Fisher Scientific) for clonal selection and expansion. Deletion was verified by means of sequencing using genomic DNA and RT-PCR, using RNA, as described recently [29], and was additionally confirmed by Western Blotting.

### RNA isolation & reverse transcription PCR (RT-PCR)

RNA was extracted from whole cell lysates using the RNeasy Plus Micro Kit (Qiagen, Hilden, Germany) and subjected to reverse transcription, followed by RT-PCR, agarose gel electrophoresis and sequencing, as described [25]. *TRPV2* expression was examined using a primer pair (forward: 5’-CCAGCAAGTACCTCACCGAC-3’; reverse: 5’-CAGGCATTGACTCCGTCCTT-3’) yielding a product sized 100 bp, as described before [15, 30]. To test for successful depletion of the above defined segment of TRPV2, oligonucleotide primers flanking the CRISPR/Cas9 deletion side of TRPV2 had the following sequences: 5’-ATGGAGGGACAGGAGGACGA-3’ (forward) and 5’-GCCATTCTCCATCTCCAGGA-3’ (reverse). In normal, TRPV2-expressing KGN cells (WT), the resulting product had an amplicon size of 290 bp, and was reduced by 117 bp in the TRPV2-deficient clones. As negative controls, cDNA synthesis reaction without enzyme (-RT) and water instead of cDNA (H_2_O) were used.

### Protein isolation & Western blotting

Isolation and processing of protein from whole cell lysates and Western Blotting were performed as described recently [15]. Briefly, 10–15 µg protein were separated in a 10–15% SDS-PAGE and transferred to nitrocellulose membranes (Amersham™ Protran™, 0.45 µM; Thermo Fisher Scientific), which were incubated overnight at 4 °C with primary polyclonal rabbit IgG antibodies targeted either against TRPV2 (1:400; Atlas Antibodies), VDAC1 (Proteintech), cleaved Caspase 3 or 8 (clCasp3, #9664T, 1:1,000; clCasp8, #98134T, 1:1,000; Cell Signaling Technology). As internal loading control, a primary monoclonal mouse IgG antibody targeted against 𝛽-Actin (A5441, 1:5,000; Sigma-Aldrich) was used. Corresponding IRDyeVR secondary antibodies (800CW donkey-𝛼-rabbit, 926-32213, 1:5,000; 680RD donkey-𝛼-mouse, 926-68072, 1:5,000; Li-COR Biosciences, Lincoln, NE, USA) were used for visualization and the Odyssey CLx imaging system (Li-COR Biosciences) for detection of bands.

### Reagents & treatment of KGN cells

Prior to any treatment, KGN cells were washed once with phosphate buffered saline (PBS; Thermo Fisher Scientific), and in order to reduce potential effects of serum the newly added medium contained only 2% FCS and was supplemented with 1% P/S. After 1 h, interference of cannabinoid receptors 1 and 2 (CB1/CNR1; CB2/CNR2) was circumvented by administration of specific blockers, AM251 (80 nM; Tocris Bioscience, Bristol, UK) and AM630 (800 nM; Tocris Bioscience), respectively as described [15, 30]. Moreover, in additional experiments interaction between CBD and the mitochondrial permeability transition pore (mPTP), most likely via VDAC1, was eliminated by using the mPTP formation blocker cyclosporin A (CysA; 7 µM; Thermo Fisher Scientific) together with AM251 and AM630. After another hour (total pre-incubation time 2 h), CBD in different concentrations (1–30 µM) or the solvent control EtOH in equivalent volume were added for either 6–24 h.

### ATP assay

To assess cell viability upon exposure to CBD, KGN cells (1 × 10^4^ cells/well) were seeded in white 96-well plates and allowed to settle overnight. The day after, cells were washed once with PBS and then pre-treated with reduced medium, supplemented with CB1 and CB2 receptor blockers alone or in combination with CysA, as mentioned above. After this 2 h pre-incubation, CBD was added to final concentrations of 1 µM, 5 µM, 10 µM, 15 µM and 30 µM for IC_50_ determination or to a final concentration of 7 µM in case of CysA co-application. The solvent EtOH served as control and was always equally adjusted to the highest volume applied (30 µM ≙ 2 µl/ml). Luminescence was measured after 6–24 h in accordance to the manufacturer’s instructions (CellTiter-Glo^®^ Luminescent Cell Viability Assay; Promega) using a microplate reader (FLUOstar; BMG labtech, Ortenberg, Germany).

### Cell counting & cell size measurement

The CASY^®^ Cell Counter (OLS OMNI Life Science, Bremen, Germany) was used to determine the cell number, used as a parameter for proliferation (untreated cells) and survival rate (CBD treated cells), and the cellular diameter of trypsinized KGN cells, as described earlier [25]. Moreover, microscopical images (hardware: Leica DM IL LED with Leica DFC 3000 G camera; software: Leica Application Suite X, version 3.7.0.20979; Leica, Wetzlar, Germany) captured after different time points were used to measure the two-dimensional, attached cell size. For each cell type, 10 cells were randomly selected from 4 representative images captured during culture (*n* = 40, each, respectively) and analyzed by means of Fiji area measurement.

### Ca^2+^ imaging & membrane potential measurement

To evaluate responsiveness of KGN cells to various compounds, Ca^2+^ imaging and membrane potential measurement experiments were performed, as described [15, 30, 31]. Briefly, a total of 1.5 × 10^5^ cells were seeded in a 35 mm imaging µ-dish (µ-Dish^35 mm, low^ with polymer coverslip; ibidi, Gräfelfing, Germany) the day prior to experimental use and were kept under standard culture conditions. Treatment was carried out as described with minor changes. For the last 30 min of pre-incubation with CB1 and CB2 receptor blockers, the cells were loaded with the Ca^2+^-sensitive fluorescence dye Fluoforte^®^ (5 µM; Enzo Life Sciences, Lörrach, Germany) or the fluorescent slow-response voltage dye DiBAC_4_(3) (500 nM; Sigma-Aldrich). This dye enables semi-quantitative measurements of the membrane potential [32]. After removal of the dye and one washing step, DMEM/F-12 supplemented with 500 nM DiBAC_4_(3) (in case of membrane potential measurement for equilibration), AM251, AM630, 2% FCS and 1% P/S was added and the dish was transferred to the imaging setup, described in more detail elsewhere [15]. The following compounds were acutely applied to the cells: CBD (1–30 µM), solvent control EtOH (equal volume to CBD); Carbachol (1 mM; Sigma-Aldrich) or H_2_O_2_ (100 µM; Sigma-Aldrich) served as positive controls (not shown). In addition, experiments with pre-incubation and co-application of the TRPV2 inhibitor SET2 (10 mM; Tocris Bioscience) were performed, following the same protocol as described previously [15]. Fluorescence intensities were monitored and are expressed as relative fluorescence intensities (normalized to the starting point, t_0_) based on background subtracted arbitrary units with a pseudo-color scale (black/purple – low intracellular Ca^2+^ / relatively hyperpolarized; red/white – high intracellular Ca^2+^ / relatively depolarized).

### Live cell Imaging, confluency & velocity measurement

Live cell imaging was used to observe the cellular consequences of CBD application and to evaluate changes in overall confluency and single-cell velocity of KGN cells based on a scratch assay. For the former, a total of 1.5 × 10^5^ cells were seeded in a 35 mm imaging µ-dish (µ-Dish^35 mm, high^ with polymer coverslip; ibidi), and 2-2.5 × 10^4^ cells per well of a culture-insert (2 well silicone insert; ibidi) attached to the bottom of the imaging dish for the latter. Cells were kept at standard conditions and allowed to settle overnight until experimental use. Following the above-mentioned treatment or removal of the insert, dishes were transferred into a stage top incubation system (ibidi) integrated to an inverted microscope (Axiovert 135; Carl Zeiss Microscopy, Oberkochen, Germany) and images were taken every 20 min using a CCD microscope camera (ProgRes^®^ MF^cool^; Jenoptik, Jena, Germany) with the corresponding software (ProgRes^®^ CapturePro, version 2.9.0.1; Jenoptik). Confluency and velocity were measured using the cellular settlement into the 500 μm cell-free gap by means of the Fiji software [33]. The overall change of confluency over time was assessed using the Fiji plugin phase contrast microscopy segmentation toolbox, PHANTAST [34], and for determination of the velocity of individual cells the plugins *manual tracking* (ImageJ; free download from: https://imagej.net/ij/plugins/track/track.html) and *chemotaxis and migration tool* (ibidi; free download from http://www.ibidi.de/applications/ ap_chemo.html) were used.

### Co-culture of primary human granulosa cells & KGN cells

For co-culture experiments, a total of 2-2.5 × 10^4^ primary human granulosa cells (hGCs) of culture day 2 were seeded in one well and the same amount of KGN cells in the other well of the above-mentioned culture insert (ibidi). Isolation and culture of these primary cells, hGCs, is described elsewhere [15]. The use of these human cells was approved by the ethical committee of LMU (project 20–697) and patients had agreed to the use of the cells. The following day, the insert was removed, cells were washed twice with PBS, treatment was conducted as described and after application of CBD, dishes were transferred into the stage top incubation system (ibidi) for live cell imaging.

### BrdU-based cell proliferation ELISA

For quantitative determination of cell proliferation, a colorimetric ELISA detecting DNA-incorporated bromodeoxyuridine (BrdU) during proliferation (11647229001; Roche, Basel, Switzerland) was used following the manufacturer’s instructions. Briefly, a total of 5 × 10^3^ cells/well were seeded in a transparent 96-well plate (Nunc-Immuno™ 96 MicroWell; Thermo Fisher Scientific) and allowed to settle overnight. The next day, medium was replaced by fresh DMEM/F-12 containing 10% FCS and 1% P/S and additionally supplemented with 10 µM BrdU, and cells were kept under standard culture conditions for one more night to allow for proliferative BrdU uptake and incorporation. BrdU was visualized using a peroxidase reaction and the optical density was measured at 380 nm in a microplate reader (FLUOstar; BMG labtech).

### Steroid measurement

To assess the impact of TRPV2 on the steroidogenic ability of KGN cells, supernatants of untreated KGN cells (*n* = 3, each) were collected after 24 h and stored at -20 °C until further processing. Samples were analyzed using liquid chromatography-tandem mass spectrometry (LC-MS/MS) at the Institute of Clinical Chemistry and Laboratory Medicine, University Hospital and Faculty of Medicine Carl Gustav Carus, Technische Universität Dresden, Dresden, Germany. Corresponding protein concentration was used for normalization, as described recently [35].

### Macropinocytosis assay

KGN cells were detached using trypsin-EDTA (PAN-Biotech, Aidenbach, Germany). Carbomer was prepared as previously described [36] using DMEM/F-12 supplemented with 10% FCS and 1% P/S. Cells were counted and adjusted to 10^5^ cells per 4 µl. For 2D assays, cells were seeded into 1 ml of fresh medium in a 6-well plate. For 3D assays, per assay 4 µl cell suspension were added to 400 µl of 0.6% carbomer hydrogel in a 1.5 ml reaction tube and incubated for 1 h. After incubation, 600 µl medium was removed from the 2D cells, 4 µl 70 kDa FITC-dextran (100 mg/ml; Invitrogen, Carlsbad; CA, USA) was added and the cells were incubated for 15 min. Dextran uptake was stopped by adding 3 ml of ice-cold EDTA solution (10 mM; Sigma-Aldrich) for 2D or by dissolving carbomer in 3 ml of ice-cold EDTA solution for 3D. After detachment with a scraper, cells were washed twice with ice-cold PBS, fixed with formaldehyde (Sigma-Aldrich) and analyzed by flow cytometry (CytoFLEX S B75408; Beckman Coulter, Brea, CA, USA).

### Immunoprecipitation & mass spectrometry

Total protein lysates were prepared from approximately 1–2 × 10^6^ KGN cells using RIPA buffer (#9806S; Cell Signaling Technology) supplemented with pierce protease and phosphatase inhibitors (A32959; Thermo Fisher Scientific), and incubated on ice for 10–15 min. The lysate was centrifuged at 16,000 *g* at 4 °C for 10 min, and the supernatant was used for immunoprecipitation (IP). IP was performed using ProteinA-sepharose beads (P9424; Millipore, Sigma-Aldrich) previously equilibrated with RIPA wash buffer (20 mM Tris, pH 7.5, 150 mM NaCl, 1 mM EDTA, 1 mM EGTA). For each IP reaction, 10 µl polyclonal rabbit anti-TRPV2 antibodies (Atlas Antibodies) were added to the cleared cell lysate and incubated for 30 min under rotation at 4 °C for 30 min. Then, 125 µl protein A-beads were added, and IP of target antigens was performed under light shaking motion at 4 °C for 90 min. Beads were washed twice in RIPA wash buffer and twice with 50 mM Tris, pH 8.0 and injected. Details on further processing of IP samples and whole cell lysates and on performance of LC-MS/MS analysis and data evaluation are described in the Supplementary Information. For library-free protein identification and quantification DIA-NN (v1.81) [37] in combination with the *Homo sapiens* subset from Swiss-Prot database was used. DAVID analysis [38] was performed with the following categories: biological process, KEGG and Reactome pathways as well as cluster Profiler package [39]. Data visualization was performed using R Statistical Software (v4.3.1). The mass spectrometry data were deposited to the ProteomeXchange Consortium (www.proteomexchange.org) via the Proteomics Identification Database (PRIDE) partner repository with the dataset identifiers PXD060778.

### Data analysis & statistics

Any mathematical and subsequent statistical analysis was done with Microsoft Excel (2024, Microsoft, Redmond, WA, USA) and Prism 9 (GraphPad, San Diego, CA, USA). Raw fluorescence data obtained from Ca^2+^ and membrane potential imaging experiments and raw confluency data collected from live cell imaging experiments were normalized to the starting time point (t_0_). Data collected from cell counting experiments and ATP assays were normalized to the corresponding solvent control EtOH and expressed in %. For Western Blotting, the integrated pixel density of any band was measured and the ratio to its corresponding ß-actin band was used for further analysis. For ATP assays, application of different concentrations of CBD was used to determine the dose-effect curve and IC_50_ of CBD in KGN cells. To do so, data normalized to the solvent control EtOH were interpolated using a sigmoidal, 4 parameter logistic (4PL) standard curve, and goodness of fit was evaluated with regard to the R squared value (R^2^ ≥ 0.96). Before any further analysis, data sets were checked for outliers (ROUT method, with coefficient *Q* = 1%) and Gaussian distribution (D’Agostino & Pearson and Shapiro-Wilk normality tests, with significance level 𝛼 = 0.05). Any comparison within a cell type, e.g. difference in clCasp3/ß-actin ratio in WT cells upon application of solvent control EtOH or CBD, was done using an unpaired, two-tailed *t*-test. For comparisons between the three cell types, WT cells and TRPV2-deficient clones #4 and #13, an ordinary one-way ANOVA with multiple comparisons was performed. For all these tests, the significance level 𝛼 was set to 0.05 (ns: not significant; * - *p* ≤ 0.05; ** - *p* ≤ 0.01; *** - *p* ≤ 0.001), and data are presented as mean ± standard error of the mean (SEM).

Prism 9 was also used to generate data plots. Throughout this manuscript, the color and symbol code for each cell type is consistent: WT in light gray with circles, TRPV2-deficient clone #4 in dark gray with upright triangle and #13 in black with upside down triangle, respectively. Data sets consisting of less than 10 experimental repetitions are depicted as column bar plots, such with 10 and more experimental repetitions are presented as min-to-max whisker box plots.

## Results

### TRPV2 in granulosa cell tumors

**Fig. 1:**
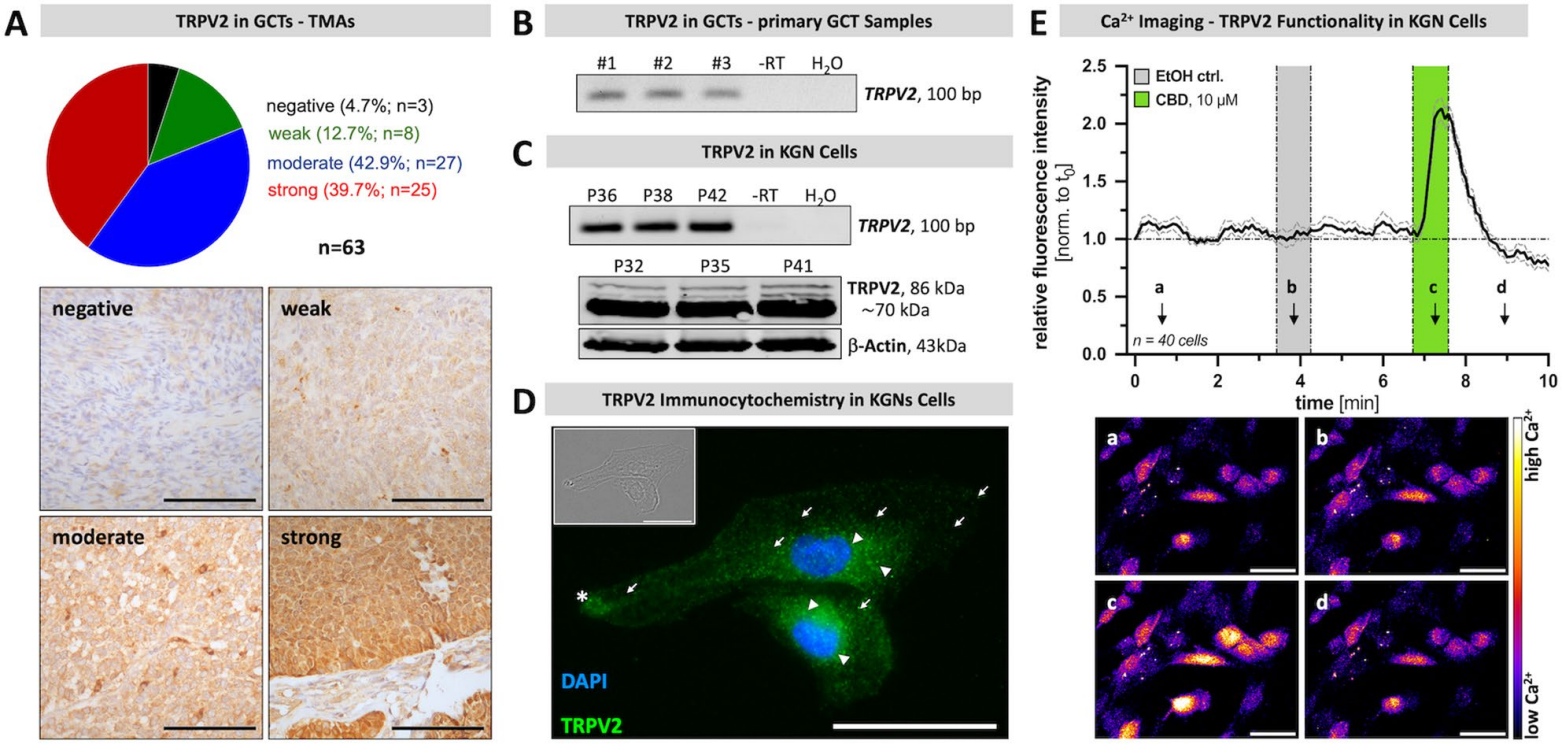
TRPV2 is expressed by GCTs and KGN cells and is functional in KGN cells.** A** TMA samples of granulosa cell tumors derived from 63 patients were subjected to immunohistochemistry targeting TRPV2 and subdivided into four categories depending on their signal intensity/density (negative (black), weak (green), moderate (blue), strong (red). Representative images of the four categories negative (upper left), weak (upper right), moderate (lower left) and strong (lower right) are shown. Scale bar 50 μm. **B** Primary GCT cells derived from three individual patients (GCT#1–3) express *TRPV2* mRNA, as demonstrated by RT-PCR. PCR amplicon size 100 bp. *-RT* (no reverse transcription) and *H*_*2*_*O* (water instead of RNA within the reaction) served as controls. Cropped gel image is shown and original gel images are shown in Supplementary Fig. 8. **C** KGN cells express TRPV2 both on mRNA (upper panel) and protein level (lower panel). PCR amplicon size 100 bp. Western Blotting revealed several bands sized between 86 kDa and ~ 70 kDa; ß-Actin served as internal loading control, band size 43 kDa. Passages used between 32 and 42 (P32-42). Cropped gel and blot images are shown and original gel images are provided in Supplementary Fig. 9. **D** Immunocytochemistry showed TRPV2 in KGN cells with fluorescence signals in close proximity to the nucleus (arrow heads), at the periphery (asterisks) or spotted and membrane-bound (arrows). TRPV2 - green; DAPI - blue; insert with phase contrast image. Scale bar 25 µM. **E** Application of cannabidiol (CBD, 10 µM; green), but not the solvent control ethanol (EtOH ctrl.; gray), elicited transient calcium (Ca^2+^) fluxes in KGN cells, as reflected by increased fluorescence intensity measured during Ca^2+^ imaging. Relative fluorescence intensity over time (normalized to starting point t_0_) of 40 examined KGN cells (mean ± SEM; mean - black line, SEM - gray dotted line), with representative live cell images displayed as pseudo-color images (black-purple - low Ca^2+^ levels; yellow-white - high Ca^2+^ levels) at the indicated time points (a-d). Scale bar 50 μm

To examine TRPV2 in granulosa cell tumors (GCTs), patient-derived GCT samples, arranged in tissue microarrays (TMAs), were subjected to immunohistochemistry (Fig. 1, A). Of the 63 GCT samples examined, 3 (4.7%) were bare of any signal, while the vast majority (95.3%; 60) exhibited expression of TRPV2 in differing intensities. A total of 12.7% (8) revealed weak signals, whereas 42.9% (27) of the samples showed moderate and 39.7% (25) strong immunoreactive signals.

Micrographs showing examples of GCTs with absence of TRPV2, weak to moderate and strong staining intensities are shown in Supplementary Fig. 1, which also shows the expression of the proliferation marker Ki67 in these groups, as assessed by a trained pathologist. Furthermore, age distribution of the patients, information on whether the sample was a primary or recurrent tumor and information of stages are provided. This last-mentioned information was, however, available only for 33 cases. No clear correlation between TRPV2 immunostaining and these parameters became evident, possibly due to the limited number of the TRPV2-negative samples. Moreover, *TRPV2* transcripts were detected in three primary GCT samples (GCT #1–3; Fig. 1, B).

### TRPV2 in KGN cells

Since studies with patient-derived primary GCT cells are limited (due to the small number of cells, high variability, partially unknown or incomplete medical history, restricted time-window for experimental use of cells), we made use of the well-established human granulosa-like tumor cell line KGN [22]. This cell line expresses TRPV2 both on transcript (upper panel) and protein level (lower panel; Fig. 1, C). Immunocytochemistry revealed TRPV2 in the perinuclear region (arrow heads), most likely representing the endoplasmic reticulum, or at spotty membranous localizations (arrows). Double immunofluorescence labeling studies supported cellular co-localization of TRPV2 with the endoplasmic reticulum marker calreticulin. Likewise partial co-localization with VDAC1 became evident (Supplementary Fig. 2). Intense TRPV2 staining of KGN cells was frequently observed at the periphery of the cell, either in a front- or ring-shaped arrangement (asterisk; Fig. 1, D). TRPV2 channel functionality was assessed upon administration of the preferential interaction partner CBD using live cell Ca^2+^ imaging (Fig. 1, E). Whereas the vehicle control EtOH (gray) had no effect on intracellular Ca^2+^ levels, acute application of 10 µM CBD (green) increased intracellular Ca^2+^ transiently, with a peak 1.8 ± 0.2-fold increase of relative fluorescence intensity (*n* = 120 cells from 3 independent experiments). The TRPV2 blocker SET2 (10 µM) inhibited the signals induced by CBD, when used at 10 and 30 µM (Supplementary Fig. 4).

### TRPV2-deficient KGN cells show significant changes in their proteome

Due to the unsatisfying pharmacological situation encompassing any TRPV2 study, we generated TRPV2-deficient KGN clones using the CRISPR/Cas9 technique, as described earlier [29]. Two out of three clones, clone #4 and clone #13, were used for further in-depth cellular studies. Sequencing confirmed the deletion, RT-PCR followed by gel electrophoresis yielded products with the correct corresponding size reflecting the size of deletion (Supplementary Fig. 3, A-D).

**Fig. 2:**
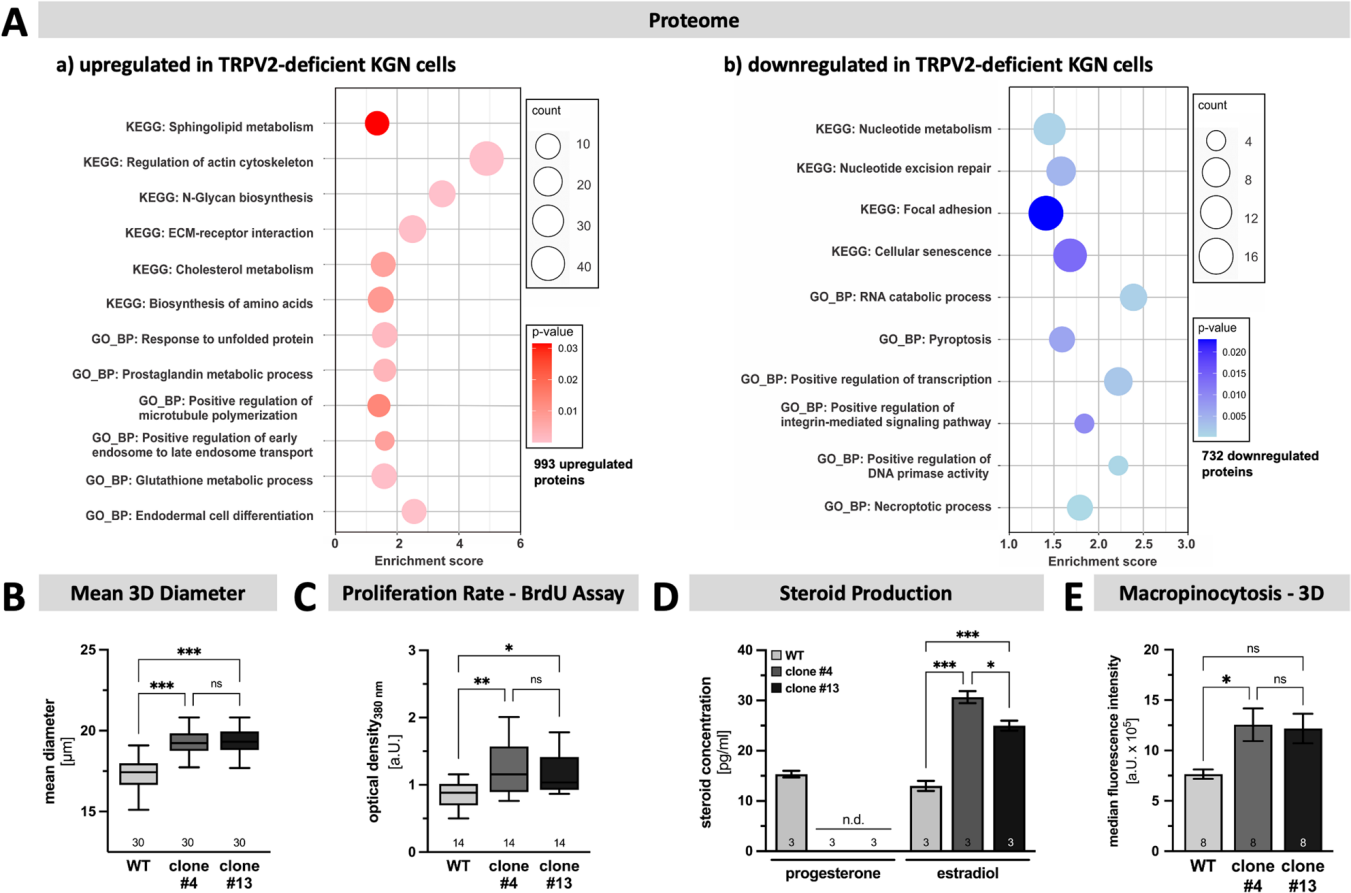
Altered proteome, increased cell size, proliferation rate, higher estradiol levels and macropinocytosis rate in TRPV2-deficient KGN cells.** A** Whole cell lysates from WT and TRPV2-deficient clone #4 subjected to mass spectrometry revealed massive changes of the proteome composition. A total of 993 proteins were upregulated, while 732 proteins were downregulated in TRPV2-deficient clones. As such, proteins related to regulation of actin cytoskeleton, but also sphingolipid and cholesterol metabolism are upregulated (a), whereas proteins associated with focal adhesion, pyroptosis and necroptotic process are downregulated upon depletion of TRPV2 (b). **B** The mean 3D diameter of trypsinized KGN cells is significantly increased in both TRPV2-deficient clones #4 and #13, compared to the WT (*n* = 30, each). **C** Proliferation rate, determined by the incorporation of BrdU 48 h after cell seeding, revealed a significant increase upon depletion of TRPV2. Data are expressed as optical density at 380 nm in arbitrary units (a.U.; *n* = 14, each). **D** Impact of TRPV2 upon steroid production in KGN cells was assessed by subjecting supernatants derived from WT and TRPV2-deficient KGN cells to liquid chromatography-tandem mass spectrometry (*n* = 3, each). Progesterone was only detected in supernatants from WT cells (n.d. - not detected), whereas the levels of estradiol were significantly higher in supernatants derived from TRPV2-deficient cells. **E** Importance of TRPV2 for macropinocytosis was assessed by measuring the uptake of 70 kDa FITC-dextran under 3D conditions. The TRPV2-deficient clone #4 engulfed significantly more than the other two cell types (*n* = 8, each)

To examine consequences of TRPV2 deletion, normal, TRPV2-expressing KGN cells (WT) and TRPV2-depleted clone #4 were subjected to mass spectrometry. Comparison of identified proteomes verified the depletion of TRPV2 and revealed alterations of cellular proteins, with a total of 993 proteins being up- and 732 proteins being downregulated, respectively. DAVID analysis (Fig. 2, A) indicated significantly upregulated expression levels of proteins related, amongst others, to regulation of the actin cytoskeleton, positive regulation of microtubule polymerization, ECM-receptor interaction, and prostaglandin metabolic processes in TRPV2-deficient KGN cells (Fig. 2, A **- **a). With significantly decreased abundance of mixed lineage kinase domain like pseudokinase (MLKL), baculoviral IAP repeat-containing protein 2 (BIRC2) and caspases 1, 4 and 6 (CASP1, 4 and 6), proteins involved in necroptotic or pyroptotic cell death, depletion of TRPV2 thus may render these cells less susceptible to cell death. Moreover, proteins linked to focal adhesion and cellular senescence showed a lower abundance in TRPV2-deficient KGN cells compared to WT cells (Fig. 2, A **- **b).

### TRPV2-deficient KGN cells feature an increase in cell size, proliferation rate, motility, steroid production and macropinocytosis

Cellular consequences of TRPV2-deletion were further assessed. The loss of TRPV2 resulted in a significantly increased 2D size of attached cells (Supplementary Fig. 5, A). With a mean covering area of 2,045 ± 94.8 µm^2^ in WT cells (Supplementary Fig. 5, A - a), the covered area by TRPV2-deficient clones, 3,825 ± 176.3 µm^2^ for clone #4 (Supplementary Fig. 5, A, b) and 3,090 ± 145.1 µm^2^ for clone #13 (Supplementary Fig. 5, A - c), was significantly higher. In the de-attached state, the increases of the mean 3D diameters (Fig. 2, B) of both TRPV2-deficient clones (#4: 19.3 ± 0.2 µM; #13: 19.4 ± 0.1 µM) were significantly increased in comparison to the WT (17.3 ± 0.2 µM).

Cell proliferation was evaluated by cell counting (Supplementary Fig. 5, B) and by BrdU incorporation (Fig. 2, C). 24 h after seeding, 31.5 ± 3.1% more WT cells were counted. This was statistically indistinguishable from the TRPV2-deficient clone #13 (increase in cell counts of 41.7 ± 3.3%). However, the TRPV2-deficient clone #4 revealed a significantly higher proliferation rate (61.7 ± 6.2%; *n* = 18, each) compared to the WT cells. Furthermore, the uptake of BrdU 48 h after seeding was significantly increased in both TRPV2-deficient KGN clones (clone #4: 1.250 ± 0.104 a.U.; clone #13: 1.154 ± 0.076 a.U.; *n* = 14, each) in comparison to WT cells (0.865 ± 0.054 a.U.; Fig. 2, C).

WT and TRPV2-deficient KGN cells differed qualitatively and quantitatively in steroid production. Using LC-MS/MS (Fig. 2, D), only progesterone and estradiol were detectable in the supernatants. Progesterone was present only in supernatants derived from WT cells (15.3 ± 0.667 pg/ml). Estradiol was detected in all supernatants but in significantly higher concentrations in the TRPV2-deficient clones (WT: 13.0 ± 1.0 pg/ml; #4: 30.7 ± 1.2 pg/ml; #13: 25.0 ± 1.0 pg/ml; *n* = 3, each).

Macropinocytosis is a receptor-independent form of endocytosis that allows cancer cells to maintain their vitality in a nutrient and growth factor limited environment by internalizing extracellular proteins and debris that can then be recycled and used as cellular fuel [40, 41]. To investigate if the loss of TRPV2 alters the macropinocytosis rate, WT cells and TRPV2-deficient clones kept under 2D and 3D conditions were fed with 70 kDa FITC-dextran and fluorescence was measured by means of flow cytometry. In 2D (Supplementary Fig. 5, C), there was a tendency of higher fluorescence intensities within the TRPV2-deficient KGN cells, but only clone #13 reached a significantly higher level in comparison to the WT cells. Cultivation in a 3D environment (Fig. 2, E) overall increased the uptake of dextran and thus the measured fluorescence intensities, compared to 2D conditions, in all three cell populations. The comparison between WT cells and TRPV2-deficient clone #4 yielded a significant difference (WT: 7.6 ± 0.5 × 10^5^ a.U.; clone #4: 12.2 ± 1.5 × 10^5^ a.U.; *n* = 8, each), whereas no other comparison showed any statistical difference.

**Fig. 3:**
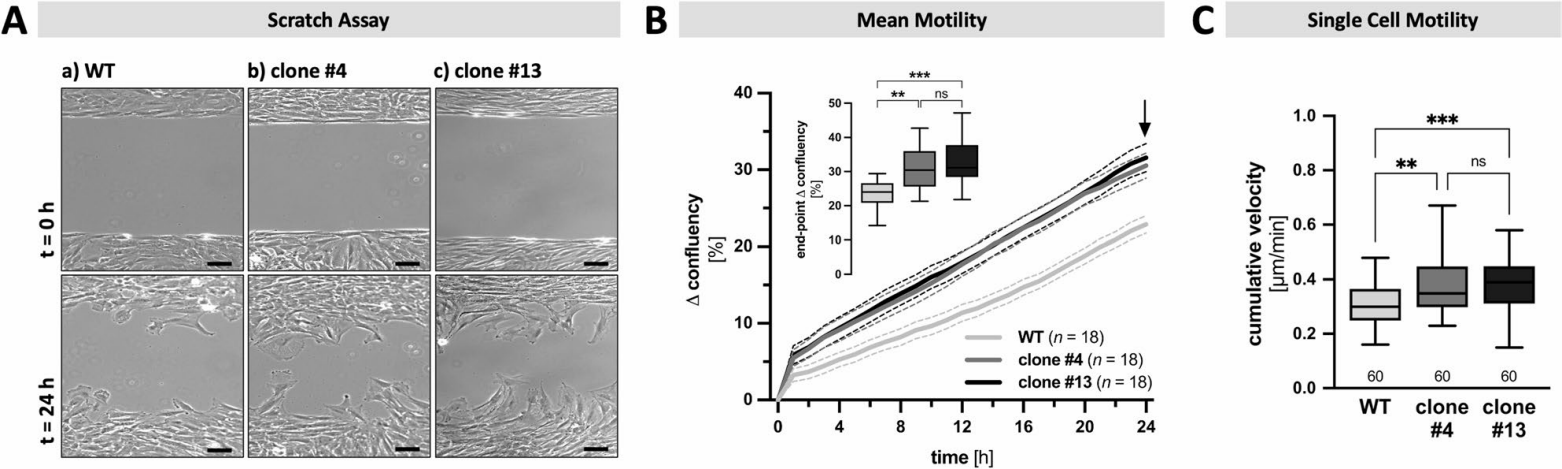
TRPV2-deficient KGN cells migrate faster.** A** Representative original micrographs from WT (a) and both TRPV2-deficient clones #4 (b) and #13 (c) captured at the beginning (upper panel; t = 0 h) and the end (lower panel; t = 24 h) of a 24 h scratch assay experiment. Scale bar 100 µM. **B** Mean motility as overall change in confluency (mean ± SEM; mean - solid line, SEM - dotted line) was analyzed using micrographs captured every 60 min for 24 h (*n* = 18, each). Inset shows changes in confluency after 24 h (end-point) revealing significantly higher efficiency in gap closing in both TRPV2-deficient clones, compared to WT cells. **C** Single cell tracking was performed to determine the cumulative velocity on single cell level for each cell type (*n* = 60, each), and revealed significantly higher cumulative velocity in both TRPV2-deficient KGN cells, compared to WT cells

To assess, if the altered abundances of proteins related to focal adhesion, the regulation of the actin cytoskeleton and microtubule polymerization may have an impact on cellular motility, scratch assay experiments were performed. Micrographs were captured every 60 min for 24 h. It became clear that the closure of the standardized gap (Fig. 3, A & B) occurred significantly faster in the TRPV2-deficient clones #4 (b) and #13 (c), respectively, compared to WT cells (**a**). In WT cells 22.9 ± 1.2% of the gap was closed after 24 h (inset), while the two TRPV2-deficient clones covered 30.6 ± 1.6% and 31.6 ± 1.9%, respectively. Next, 60 single cells of each cell line were tracked over time. The analysis revealed a mean cumulative velocity of 0.307 ± 0.01 μm/min for WT cells. TRPV2-deficient cells showed a significantly increased velocity of 0.380 ± 0.01 μm/min for clone #4 and 0.379 ± 0.01 μm/min for clone #13, respectively (Fig. 3, C). Representative trajectory plots demonstrate the difference between WT and TRPV2-deficient cells in terms of distance covered within 24 h (Supplementary Fig. 5, D).

### CBD induces apoptotic cell death in KGN cells in a dose and time dependent manner

**Fig. 4:**
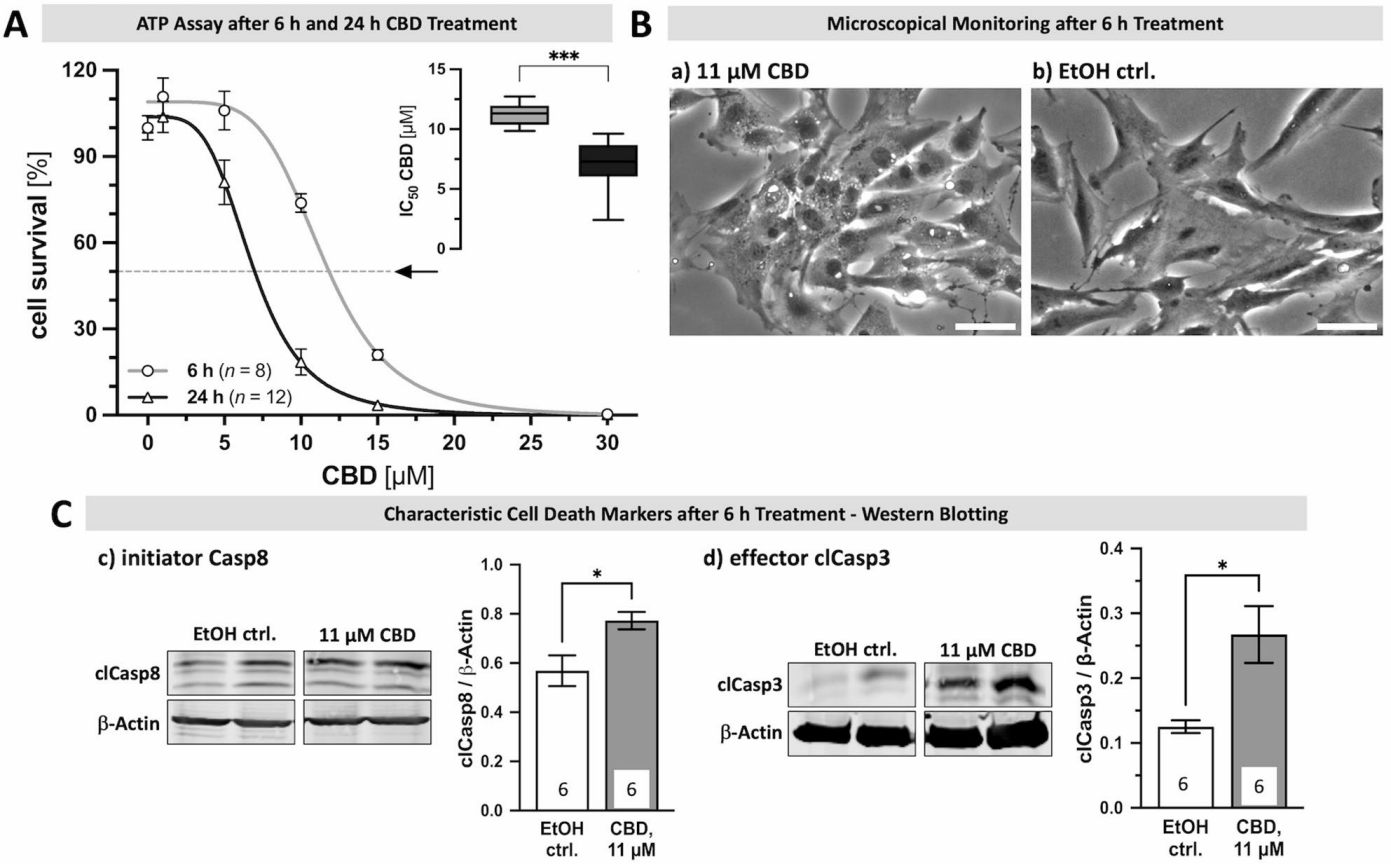
KGN cells are susceptible to CBD-induced cell death.** A** Application of CBD in different concentrations (1 µM, 5 µM, 10 µM, 15 µM and 30 µM) or the solvent control EtOH for 6 h (gray line with circles; *n* = 8) and 24 h (lead line with triangles; *n* = 12) and measurement of cell survival (in % to solvent control EtOH) by means of ATP assays was used to determine the CBD IC_50_ for KGN cells. Results are shown as interpolated dose-effect curves based on identified mean ± SEM cell survival for each concentration applied. Inset depicts variance of interpolated CBD IC_50_ values for 6 h (gray) and 24 h incubation (lead) of all performed experiments. **B** Representative micrographs captured after 6 h treatment with 11 µM CBD (a) or the solvent control EtOH (b; EtOH ctrl.). Presence of CBD induced first signs of cell death, such as intracellular vacuolization and nuclear condensation, while the solvent control EtOH had no effects on cellular morphology or appearance. Scale bar 50 µM. **C** Treatment of KGN cells with 11 µM CBD for 6 h resulted in increased levels of the characteristic apoptotic cell death markers cleaved (cl) initiator caspase (Casp) 8 (c) and effector clCasp3 (d), as identified by means of Western Blotting. β-Actin served as internal loading control. Representative bands from KGN cells treated either with the solvent control EtOH or 11 µM CBD, respectively. Cropped blot images are shown; original gel images provided in Supplementary Fig. 9

As the proteomic data indicated roles of TRPV2 in cell death, and because several reports have described such a link [32, 42, 43], we examined cytotoxic effects of channel activation in KGN cells, making use of the preferred activator CBD, in lack of a more specific agonist. Different concentrations of CBD were applied to WT cells, for 6–24 h, and cell viability was determined by ATP assays (Fig. 4, A). At a concentration of 1 µM CBD, no cytotoxic effects were observed. However, incubation with 10 µM or 15 µM CBD for 6 h strongly reduced the number of viable cells, with 73.8 ± 3.3% and 21.0 ± 1.9% of the cells still being alive (light gray line & circles; *n* = 8, each). Moreover, 24 h treatment with 5 µM, 10 µM, 15 µM or 30 µM CBD reduced the number of viable cells by 17.8 ± 7.9%, 83.7 ± 4.0%, 96.6 ± 1.0%, and 99.9 ± 0.0% (dark gray line & triangles; *n* = 12, each), respectively. Cell counting experiments yielded similar results (Supplementary Fig. 6, A). Using a sigmoidal standard curve for interpolation (4PL), an IC_50_ of 11.3 ± 0.3 µM (6 h) and 7.0 ± 0.6 µM (24 h) was defined for CBD in KGN cells (Fig. 4, A **-** inset).

In parallel, cells were microscopically monitored (Fig. 4, B) and after 6 h of incubation with 11 µM CBD (a), cellular shrinkage and withdrawal of cell projections, intracellular vacuolization, nuclear condensation, and cellular ballooning were observed. The solvent control EtOH had no such effects (Fig. 4, B **- **b).

To examine possible mechanisms of cell death, we first examined, whether TRPV2 channel opening upon prolonged CBD application affects the resting membrane potential, using the fluorescent slow-response voltage dye DiBAC_4_(3). Changes in fluorescence intensity in presence of the solvent control EtOH (gray) or CBD (green) were evaluated (Supplementary Fig. 6, B). While EtOH did not affect fluorescence intensity, the addition of 30 µM CBD strongly increased the signal after a few minutes, indicating depolarization. The maximum change of fluorescence intensity in presence of CBD within the dish reached a level of 181.1 ± 8.2% and the final level was 271.7 ± 13.7% (*n* = 40 cells). Moreover, with progressing imaging time a general cellular shrinkage and partial detachment occurred and first signs of ballooning became obvious.

Next, characteristic cell death markers were used to define the type of cell death occurring in KGN cells upon application of CBD by means of Western Blotting (Fig. 4, C). After treatment with 11 µM CBD for 6 h, significantly increased levels of the cleaved (cl) and thus active apoptosis-associated initiator and effector caspases (Casp) 8 and 3, respectively, were observed. The levels of clCasp8 increased 1.4-fold (**c**), and that of clCasp3 2.1-fold (**d**).

### KGN cells are vulnerable to CBD-induced cell death but not primary hGCs

Primary hGCs are also known to express functional TRPV2 [15] but did not show signs of cell death in the presence of CBD at a concentration of 7 µM (24 h IC_50_ identified for KGN cells). In order to test if CBD-induced cell death is indeed exclusive for KGN cells, both cell types were co-cultured and treated with 7 µM CBD for 24 h in a live cell imaging setup (Supplementary Fig. 6, C). The primary hGCs tolerated both the presence of 7 µM CBD (**c**) and the solvent control EtOH (**d**) and did not show signs of cell death but rather spread on the surface (hGCs boxed in cyan, left half of the optical view), whereas KGN cells rapidly showed signs of cell death with withdrawal of cell projections, intracellular vacuolization, ballooning and detachment (KGNs boxed in pink, right half of the optical view). As observed in all other experiments, the solvent control EtOH had no effect on cellular appearance and did not induce cell death in KGN cells, which instead proliferated, spread on the surface and moved towards the hGC compartment.

### Depletion of TRPV2 makes KGN cells less sensitive to CBD and CBD-induced changes in membrane potential and cell death

**Fig. 5:**
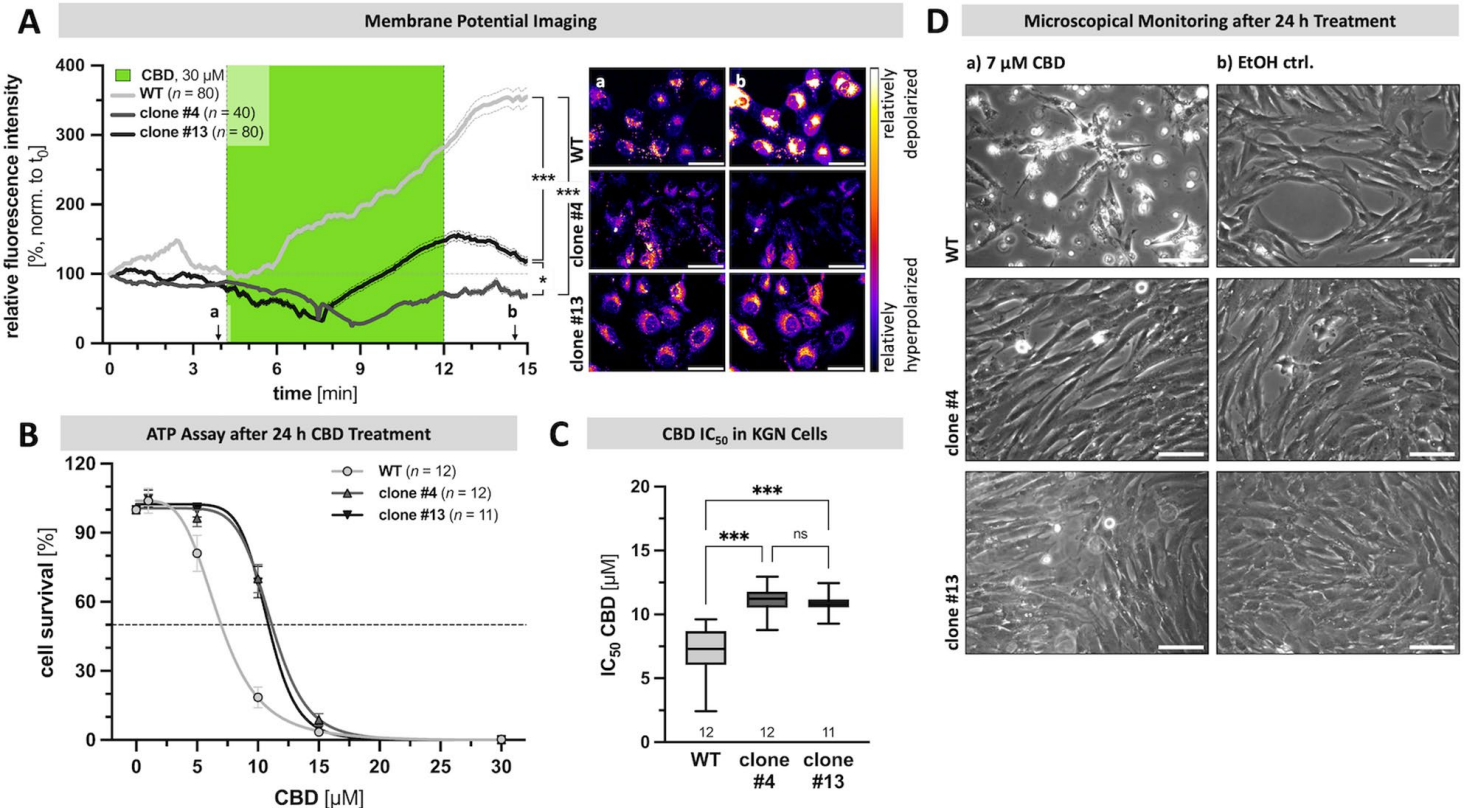
Absence of TRPV2 makes KGN cell less vulnerable to CBD-induced depolarization and cell death.** A** Presence of CBD (30 µM; green) strongly increased the resting membrane potential of WT cells (light gray; *n* = 80 cells), whereas the TRPV2-deficient clones #4 (gray; *n* = 40 cells) and #13 (dark gray; *n* = 80 cells) were significantly less sensitive to such changes (statistical comparison the end point intensities), as demonstrated by means of membrane potential imaging experiments based on emission of the voltage sensitive fluorescence dye DiBAC4(3) (mean ± SEM; mean - black line, SEM - gray dotted line). Relative fluorescence intensity over time (normalized to starting point t_0_), with representative live cell images of WT (top panel), and TRPV2-deficient clones #4 (middle panel) and #13 (bottom panel), respectively, displayed as pseudo-color images (black-purple – relatively hyperpolarized; yellow-white – relatively depolarized) at the start (a) and end of CBD presence within the dish (b). Scale bar 50 μm. **B** Consequences of application of the solvent control EtOH or different concentrations of CBD (1 µM, 5 µM, 10 µM, 15 µM and 30 µM) for 24 h on cell survival of WT cells (*n* = 12) and TRPV2-deficient clones #4 (*n* = 12) and #13 (*n* = 11), respectively, were assessed by ATP assays. Results are shown as interpolated dose-effect curves based on all performed experiments. **C** Corresponding interpolated CBD IC_50_ values for WT and TRPV2-deficient KGN cells revealed significantly higher concentrations needed to kill 50% of cells in absence of TRPV2. **D** Representative micrographs of WT (top) and TRPV2-deficient clones #4 (middle) and #13 (bottom) captured at the end of a 24 h treatment with either 7 µM CBD (a) or the solvent control EtOH (b), revealing the occurrence of cell death only in WT cells. Scale bar 100 μm

To test if TRPV2 deficiency influences CBD sensitivity and vulnerability to cell death in KGN cells, both WT and TRPV2-deficient KGN cells were treated with CBD. At the same time, the resting membrane potential was monitored for 15 min (Fig. 5, A), cytotoxicity was assessed after 24 h by ATP assays (Fig. 5, B &C), and in addition cells were microscopically monitored (Fig. 5, D).

While 30 µM CBD (green) rapidly increased the resting membrane potential of WT cells by 354.5% (*n* = 80 cells), the resting membrane potential of both TRPV2-deficient clones was significantly lower and either even slightly decreased (#4: 69.3%, *n* = 40 cells) or increased (#13: 107.8%, *n* = 80 cells) with regard to their corresponding starting levels (Fig. 5, A). Changes in fluorescence intensities at the indicated time points (a & b) are highlighted by representative micrographs of each cell line (right panel).

The loss of TRPV2 also resulted in a significantly reduced vulnerability to CBD-induced cell death. While CBD induced cell death in WT cells with an IC_50_ of 7 µM (24 h treatment), this concentration caused only limited cell death in TRPV2-deficient KGN cells, with 16.8 ± 4.5% in clone #4 (*n* = 12) and 15.8 ± 4.3% in clone #13 (*n* = 11). Treatment with different concentrations of CBD (1–30 µM) for 24 h followed by measurement of cell viability / cytotoxicity revealed a clear shift of the cell death-inducing CBD IC_50_ to higher values (Fig. 5, B). As such, for TRPV2-deficient KGN cells a CBD concentration of 11.1 ± 0.3 µM and 10.9 ± 0.3 µM was required to kill half of the seeded cells of clone #4 or #13, respectively (Fig. 5, C).

Microscopical monitoring strongly supports this observation. WT cells showed the above-described signs of cell death with intracellular vacuolization and nuclear condensation after 6 h treatment with 11 µM CBD (Supplementary Fig. 7, A - a), and moreover were almost completely de-attached from the dish after a 24 h treatment with 7 µM CBD (Fig. 5, D - c). In contrast, both the TRPV2-deficient clones #4 (d) and #13 (e) were not visibly affected and the dishes were almost confluent, both after 6 h treatment with 11 µM CBD (Supplementary Fig. 7, A - a) and 24 h treatment with 7 µM CBD (Fig. 5, D - c), respectively. At any of these two time points, the solvent control EtOH had no effect on cell viability or morphology of any cell type (Fig. 5, D - d & Supplementary Fig. 7, A - b).

However, higher concentrations of CBD (15 µM and 30 µM tested) abolished statistical differences and cell survival rates were comparably low in both WT cells and TRPV2-deficient clones. This may indicate that some CBD actions are independent of TRPV2. Indeed, CBD is known to have multiple molecular targets [44, 45].

### TRPV2 interactome in KGN cells

**Fig. 6:**
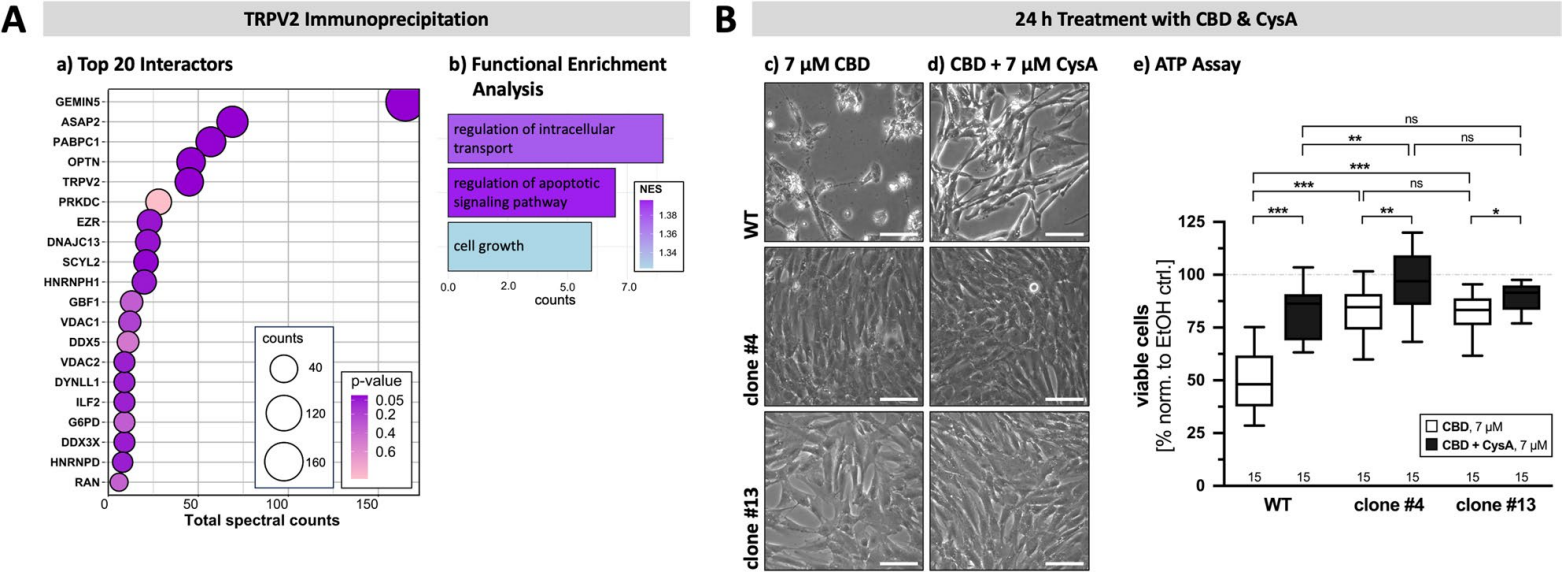
TRPV2 interactome in KGN cells and involvement of the mPTP in CBD-induced cell death.** A** TRPV2 interactome in KGN cells identified by immunoprecipitation followed by mass spectrometry. Top 20 of identified interactors (a) that can be categorized into three functional groups, i.e. regulation of intracellular transport, the regulation of apoptotic signaling pathway and cell growth, in accordance with a functional enrichment analysis. Note that VDAC1 and VDAC2 are among the top identified proteins and may be potential interactors. (b). **B** Impact of the mPTP formation blocker CysA (7 µM) on cytotoxic effects of CBD (7 µM) was assessed by means of microscopic monitoring (c & d) and ATP assays (e). Representative micrographs of WT (top) and TRPV2-deficient clones #4 (middle) and #13 (bottom) captured at the end of a 24 h treatment with either 7 µM CBD alone (c) or 7 µM CysA alongside with 7 µM CBD (d), revealing the occurrence of cell death only in WT cells whilst TRPV2-deficient clones appear healthy. CysA featured a protective effect on this CBD-induced cell death in WT KGN cells. Scale bar 100 μm. ATP assays quantitatively support this observation and confirmed the highly significant protective effect of CysA on CBD-induced cell death in all three cell types (*n* = 15, each)

Next, we examined the interactome of TRPV2 in KGN cells. Immunoprecipitation pull-down experiments were performed and interacting proteins were identified by means of mass spectrometry (Fig. 6, A). Within the top 20 of identified proteins (a), TRPV2 interacts with proteins that are involved in membrane trafficking, i.e. SCY1 like pseudokinase 2 (SCYL2) and optineurin (OPTN) [46, 47], but also, together with ArfGAP with SH3 domain, ankyrin repeat and PH domain 2 (ASAP2), plays a role in phagocytosis and cell migration [48, 49]. The identified interactors can be categorized into three functional groups, i.e. regulation of intracellular transport, regulation of apoptotic signaling pathway and cell growth, respectively (Fig. 6, A - b).

Of note, the mitochondrial voltage-dependent anion channel 1 (VDAC1) and also VDAC2 were identified as potential TRPV2 interaction partners in KGN cells. In double immunofluorescence labeling studies partial co-localization of VDAC1 and TRPV2 became apparent (Supplementary Fig. 2). VDAC1, in turn, is a known target for CBD [50]. As shown by immunohistochemistry in tissue samples of GCT samples, VDAC1 is present in all examined GCTs with intense spotted intracellular localization most likely representing mitochondria (Supplementary Fig. 7, B - c). In WT and also in both TRPV2-deficient clones #4 and #13, respectively, immunoblotting against VDAC1 yielded a clear and strong band at the corresponding size of 31 kDa (Supplementary Fig. 7, B - d).

### Involvement of VDAC1/2 and mPTP in CBD-induced cell death in KGN cells

CBD was described to induce cell death by direct modulation of the mitochondrial voltage-dependent anion channel 1 VDAC1 in murine microglial cells [50], which could be reversed by blocking the formation of the structurally unsolved mitochondrial permeability transition pore (mPTP). Therefore, the effect of the mPTP formation inhibitor cyclosporin A (CysA) on CBD-induced cell death in KGN cells was assessed (Fig. 6, B). To this end, cells were pre-incubated with 7 µM CysA for 1 h, then treated with 7 µM CBD for 24 h followed by evaluation of cell viability by means of microscopical imaging (Fig. 6, B - c & d) and ATP assay (Fig. 6, B - e). As expected, the addition of 7 µM CBD (Fig. 6, B - c) killed and de-attached almost all WT cells (upper panel). Cells still being present and attached to the dish featured condensed nuclei and intracellular vacuolization, as observed before. At the same time, both TRPV2-deficient clones (middle and lower panel) had a healthy appearance, confluency was only slightly decreased in clone #13, respectively. In presence of CysA (Fig. 6, B -d), effects of 7 µM CBD on both TRPV2-deficient clones were not visible, while the WT cells were more resistant to CBD-induced cell death. Thus, more cells were still present and alive after 24 h treatment, although not at the same confluency level as its TRPV2-deficient counterparts. The solvent control EtOH (Supplementary Fig. 7, C) had no effect on any of the three cell types and cells appeared healthy and confluent.

Quantitatively in line with these observations, ATP assays (Fig. 6, B - e) revealed that while 50.7 ± 4.0% of WT cells survived the treatment with 7 µM CBD, the survival rate significantly increased to 82.5 ± 3.2% viable cells when CysA was co-applied to CBD. In the TRPV2-deficient clones, survival rate after sole treatment with 7 µM CBD for 24 h amounts to 82.0 ± 3.4% for clone #4 and to 82.6 ± 2.2% for clone #13. The co-application of CysA and CBD scored significant higher survival rates with 97.6 ± 3.6% and 89.4 ± 1.8% for clone #4 and #13, respectively. Of special note, while sole CBD treatment clearly revealed dependency of CBD-induced cell death on expression of TRPV2, CysA was able to level these differences, at least partially. In addition, cell counting of the same experimental setup yielded comparable results, but here CysA completely abolished any difference between the three cell types (Supplementary Fig. 7, D).

## Discussion

Despite extensive research during the last decades, TRPV2 is still regarded a “drug orphan” [4–8, 51, 52]. Consequently, the generation of TRPV2-deficient KGN cells was essential in an attempt to examine roles of this channel in tumor cells originating from the human ovary. We found that the deletion of this channel had severe consequences for KGN cells. The proteomic results revealed several regulated pathways, which provide new insights into the role of TRPV2. The proteomic changes are in line with alterations in cell behavior. Specifically, the increased proliferation rate and faster movement of TRPV2-depleted cells, imply that the absence of this channel induces a more malignant cellular phenotype, which is accompanied by altered steroid hormone profiles.

Completely TRPV2-negative GCTs were in the minority in our panel of 63 primary tumor samples, but TRPV2 expression levels varied from absent to weak and strong. It seems possible that tumors with no or weak TRPV2 expression may represent a more aggressive subgroup. Additional studies with substantially more samples and corresponding clinical data will be required to put this assumption to the test. Furthermore, it seems conceivable that such a subgroup could be distinguished and possibly detected already by its steroid hormone output. Again, additional studies are now required to follow up on these observations and determine whether they are transferable to the in situ situation. If so, TRPV2-expression may serve as novel prognostic marker of GCTs. At present time the repertoire of prognostic markers for GCTs is rather limited to three proteins, which provide a prognostic value for worse outcome, i.e. the neural cell adhesion molecule (NCAM or CD56), the transcription factor GATA-4 and the mothers against decapentaplegic homolog 3 (SMAD3) [21].

GCTs are rare malignant human tumors and next to adequate biomarker and prognostic markers, they still lack a specific treatment. Based on our study, we propose that targeting TRPV2 may be a novel option. In WT, TRPV2-expressing KGN cells, activation of TRPV2 was achieved by CBD, in lack of a more specific pharmacological activator. This preferred activator of TRPV2 [18] induced apoptotic cell death in KGN cells in a time- and concentration-dependent manner. The mechanism of action involved the rapid breakdown of the membrane potential, which was identified as an early consequence of TRPV2 activation and was absent in TRPV2-deficient clones. In the TRPV2-depleted KGN cells, cell death was significantly reduced and thereby indicated a crucial role of TRPV2 in this event.

However, especially when higher CBD concentrations were tested, CBD-induced cell death occurred also in TRPV2-depleted KGN cells. This indicates that CBD actions involve not only activation of TRPV2 but also additional targets and mechanisms.

CBD is known to possess a multitude of interaction partners at the molecular, cellular and organ level, as reviewed elsewhere [44, 45, 53, 54]. CBD has been implicated in the induction of different forms of cell death in several human tumor types previously [50, 55–61]. In a set of these studies, the underlying mechanism could be traced back to mitochondrial dysfunction. This was due to compensatory persistent opening of the mitochondrial permeability transition pore (mPTP) downstream of a CBD-dependent decrease in conductance of the voltage dependent anion channel 1 (VDAC1), finally leading to mitochondria-mediated apoptosis [50, 62–64]. VDAC1, which is regarded as a promising pharmacological target in cancer [65], is present in KGN cells and all analyzed primary GCT samples, as expected. Results of immunoprecipitation studies suggested that it could be a potential member of the TRPV2-interactome in KGN cells. The results were robust and confirmed in four independent immunoprecipitation experiments. Our immunofluorescence studies show partial co-localization of TRPV2 and VDAC1/2 in KGN cells. Yet, final conclusions about interactions of the proteins await confirmation by additional studies. Of note, irrespective of the outcome of such studies, VDAC1 is a known target of CBD. Therefore, VDAC1 being part of the TRPV2 interactome in KGN cells is not a necessity of CBD to act on TRPV2 [66, 67].

To examine whether VDAC1-associated events may be involved in cell death induced by CBD, we tested cyclosporin A, which has been described to diminish CBD-induced cell death by blocking mPTP formation [50]. It indeed significantly decreased the vulnerability of WT, TRPV2-expressing KGN cells, and TRPV2-depleted clones, to CBD-induced cell death.

Hence, some of the actions of CBD in KGN cells are direct consequences of TRPV2 activation and additional parts are related to interactions of CBD with VDAC1, downstream involving the mPTP. The results taken together indicate that TRPV2 and, most likely, VDAC1 are the two major players, which can be linked to CBD-induced cell death in KGN cells.

The results may have important implications. Next to the possibility of serving as a prognostic marker for GCTs, TRPV2 may be a novel drug target in GCTs. Further, although no specific drug activator for TRPV2 exists yet, CBD is readily available and may even be of advantage, as it targets both TRPV2 and VDAC1. Furthermore, it may spare non-tumor hGCs, in which CBD promotes an inflammatory response [15].

## Supplementary Information

Supplementary Material 1.

## Data Availability

The mass spectrometry proteomics data underlying this article have been deposited to the ProteomeXchange Consortium via the PRIDE partner repository (https://www.proteomexchange.org/) with the dataset identifier: PXD060778.
