# Supplementary material for: TRPV2 regulates cell fate in the human granulosa-like tumor cell line KGN: implications for granulosa cell tumors and cannabidiol
Source: Cell Commun Signal. 2026 Feb 12;24:139. doi: 10.1186/s12964-026-02729-y (PMC12930786; doi:10.1186/s12964-026-02729-y)
Supplement: Supplementary file 1 — Supplementary Material 1. [file 12964_2026_2729_MOESM1_ESM.docx]

**TRPV2 regulates cell fate in the human granulosa-like tumor cell line KGN: Implications for granulosa cell tumors and cannabidiol**

Katja Eubler ^1^, Sree Priyanka Jeevanandan ^1^, Karolina Magdalena Caban ^2^, Jan Bernd Stöckl ^2^, Malte Benjamin Braun ^3^, Carola Herrmann ^1^, Michaela Schneider ^1^, Nicole Kreitmair ^1^, Lina Scholz ^1^, Doris Mayr ^4^, Jörg Renkawitz ^3^, Harald Welter ^1^, Annette Müller-Taubenberger ^1^, Thomas Fröhlich ^2^, Artur Mayerhofer ^1^

^1^ Biomedical Center Munich (BMC), Cell Biology, Anatomy III, Faculty of Medicine, Ludwig Maximilian University (LMU) Munich, Planegg-Martinsried, Germany

^2^ Laboratory for Functional Genome Analysis LAFUGA, Gene Center, LMU Munich, Munich, Germany

^3^ Biomedical Center, Walter Brendel Center of Experimental Medicine, Institute of Cardiovascular Physiology and Pathophysiology, University Hospital, LMU Munich, Munich, Germany

^4^ Institute of Pathology, University Hospital, LMU Munich, Munich, Germany

**Correspondence:**

mayerhofer@bmc.med.lmu.de

[https://orcid.org/0000-0002-9388-4639](https://orcid.org/0000-0002-9388-4639%20)

**Grant support:** This study was funded by DFG project #456828204.

**Key words**: Ovary, granulosa cell tumor, cell death, biomarker, drug target

**Short title**: Cannabidiol and cell death in human granulosa tumor cells

**Supplementary Files**

**Supplementary Figure 1: Proliferation index Ki67 and TRPV2 expression, examples of TRPV2 staining of GCT samples and patient data.**

1. Ki67 expression of tumor samples (<5%; 5-10%; 11-20%) and immunoreactivity for TRPV2 (strong +++; medium/weak ++/+, negative -) in n=63 GCT samples; examples for strong (left panel), intermediate (middle panel) and absent expression (right panel) are shown

(* blood vessel).

1. Age distribution of patients with GCTs with strong (+++) and medium/weak (++/+) or negative (-) staining for TRPV2 (n.s.: not significant)
2. Intensities of TRPV2 staining (+++; ++/+/-) in primary and recurrent tumors (n=63) and correlation to tumor stages (pT1 and pT2-3) in n=33 GCT samples.


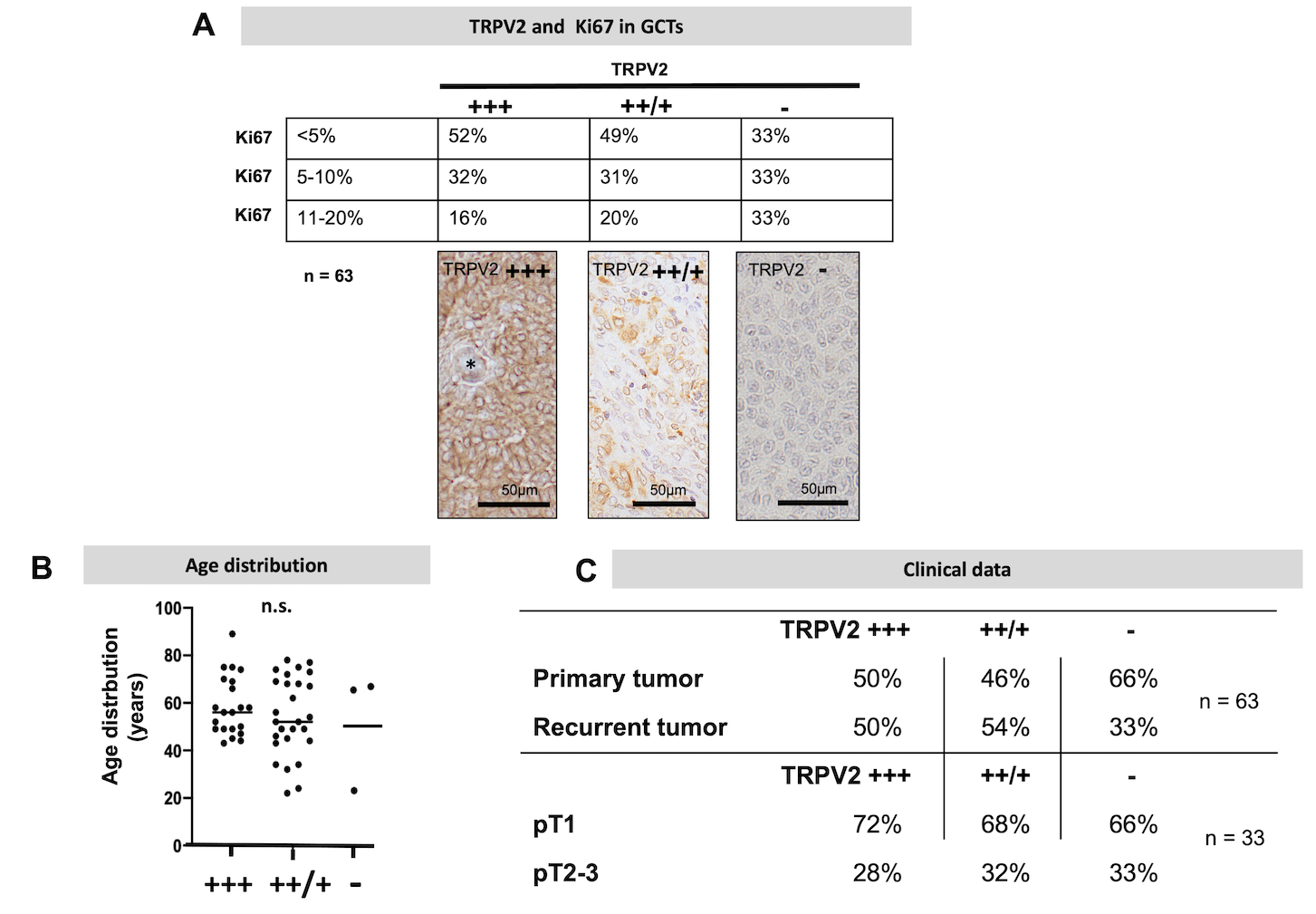


**Supplementary Figure 2: Immunofluorescence co-localization studies in KGN cells**

Immunofluorescence detection of TRPV2 (red) and the ER marker calreticulin (green); bar: 10 µm

1. Immunofluorescence detection of TRPV2 (red) and the ER marker calreticulin (green); nuclei were stained with DAPI. Single channels and merged micrograph. Scale bar 10 µm.
2. Immunofluorescence detection of TRPV2 (green) and VDAC (red); nuclei were stained with DAPI. Single channel and merged micrographs. Scale bar 10 µm.
3. **
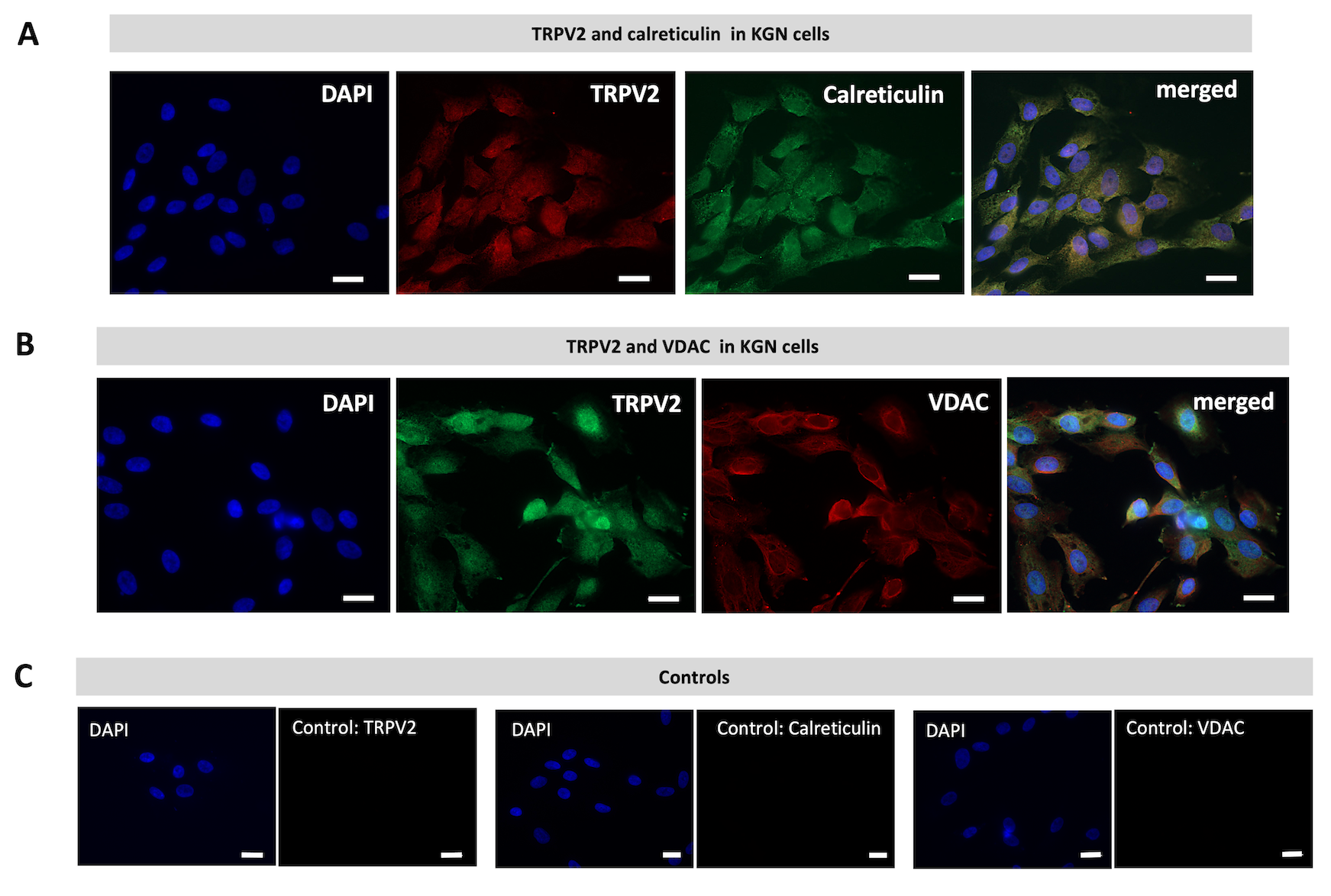
**Examples of control experiments, in which the respective primary antibody was omitted. Scale bar 10 µm.

**
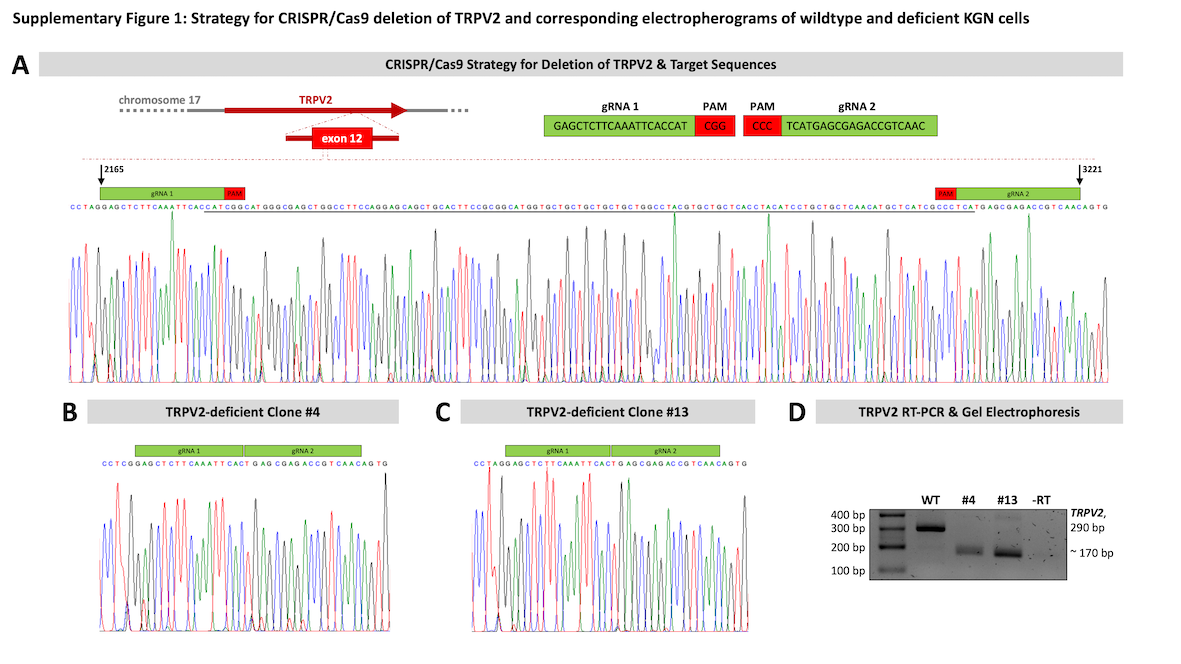
Supplementary Figure 3: Strategy for CRISPR/Cas9 deletion of TRPV2 and corresponding electropherograms of wildtype and deficient KGN cells. A**) Graphical illustration of the TRPV2 deletion site located within exon 12 of the coding region on chromosome 17 (upper panel), and the relevant sequencing section of an electropherogram of WT KGN cells with highlighted gRNA binding sites, position 2165 and 3221, and nucleotides to be deleted (underlined). **Inset** depicts gRNA sequence boxed in green and PAM sequence, three bps upstream of the target, boxed in red. **B** & **C**) Edited sequence of TRPV2-deficient clones #4 and #13, respectively. PAM sequence and three nucleotides upstream were successfully deleted. **D**) Successful deletion was initially confirmed by means of RT-PCR resulting in a product reduced in size (~290 bp band in WT, ~170 bp band in clones #4 and #13). Omission of the reverse transcriptase (-RT) during cDNA synthesis served as negative control. Cropped gel image shown, original gel images are provided in **Supplementary Figure 8**.

**Supplementary Figure 4: CBD-induced elevations of intracellular Ca^2+^ in KGN cells are blocked by the TRPV2 antagonist SET2.**

**
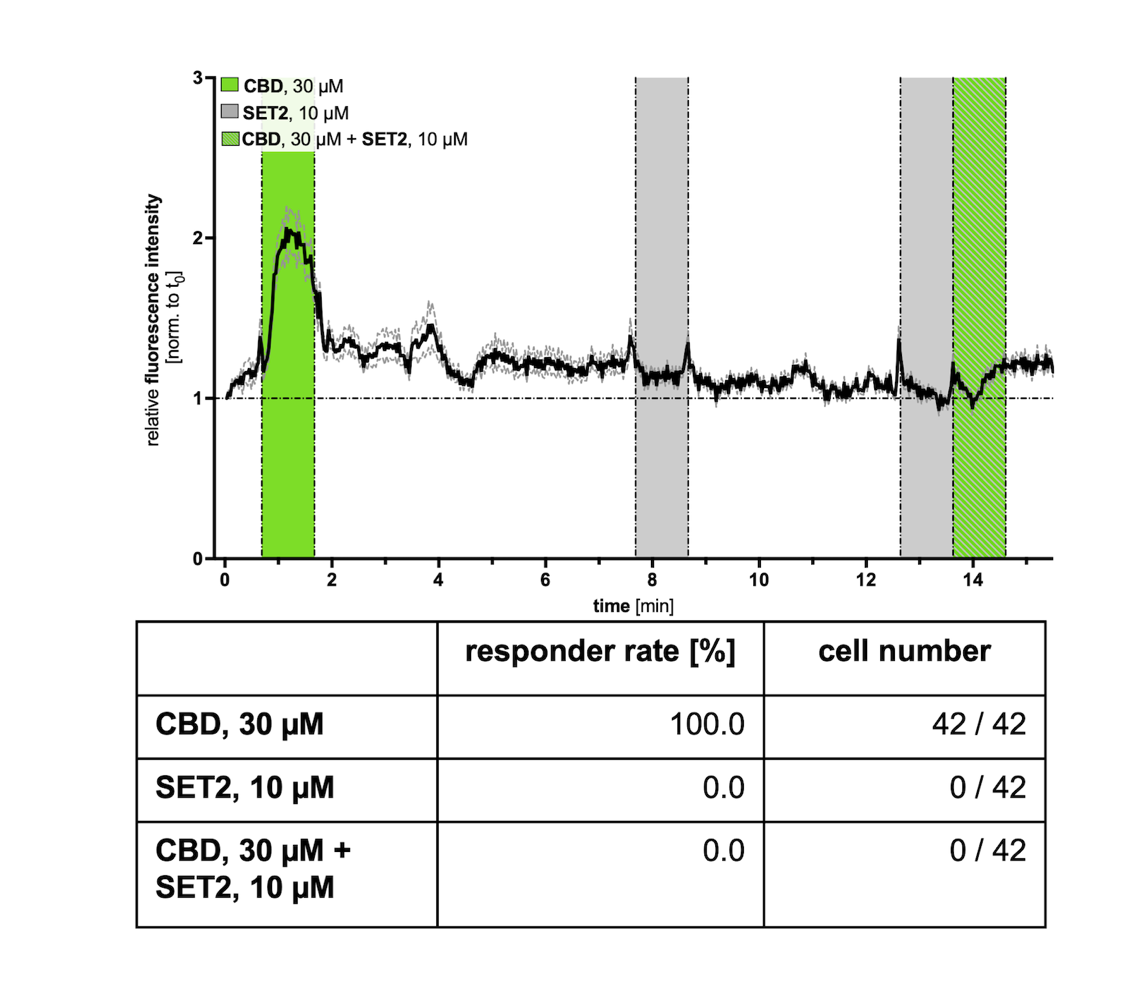
**Example of a Calcium Imaging experiment, in which we tested specificity of the observed CBD-induced Ca^2+^ transients by applying the antagonist SET2. In presence of CNR1/2 blockers (AM251 and AM630), 30 μM CBD elicited Ca^2+^ transients in all imaged cells (42/42), whereas there was no response to 10 μM SET2 (0/42). CBD did not elicit any Ca^2+^ transients during co-application with SET2. Graph shows averaged original traces (black solid line) and SEM (grey dashed line) of 11 cells. In the table responder rates and cell numbers are given.

Further experiments were performed with 10 µM CBD yielding similar results: All 30 cells examined in this experiment responded to CBD with Ca^2+^ transients and SET2 effectively blocked the increase.

**Supplementary Figure 5: Loss of TRPV2 results in increased cell size, proliferation rate and motility in KGN cells - Part 2. A)**2D cell size of normal, TRPV2-expressing KGN cells (WT) and TRPV2-deficient clones #4 and #13 were determined based on micrographs captured during culture time. Of each cell type, 40 cells were randomly selected and their area was measured, revealing a significant increase in 2D cell size of TRPV2-deficient KGN cells. **a**-**c**) Representative micrographs of each cell type highlighting the increase in 2D cell size upon depletion of TRPV2. Scale bar 100 µM. **B)**Proliferation rate was additionally determined by cell counting after 24 h and revealed significant changes upon depletion of TRPV2. Data are expressed as % of the initially seeded cell number (n = 18 for each cell type). **C**) Macropinocytosis rate of 70 kDa FITC-dextran under 2D conditions. The TRPV2-deficient clone #13 engulfed significantly more than the other two cell types (n = 8, each). **D**) Representative trajectory plots of 10 WT cells (**d**) and 10 TRPV2-deficient cells of clones #4 (**e**) and #13 (**f**), respectively, used for identification of cumulative velocity of single cells.


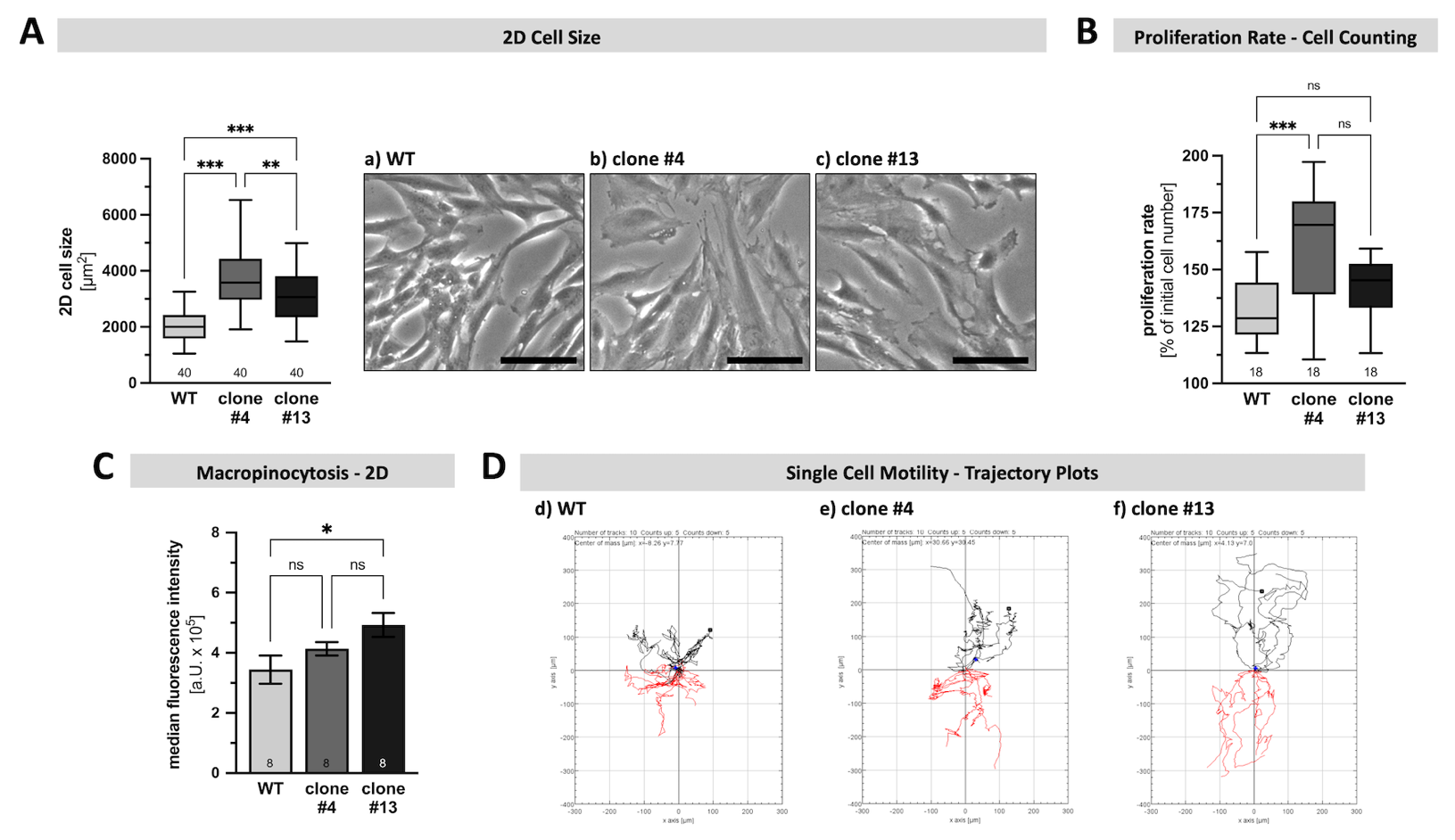


**Supplementary Figure 6: KGN cells are susceptible to CBD-induced cell death, but primary human GCs are not. A**) Cell viability of normal, TRPV2-expressing KGN cells upon application of different concentrations of CBD (1-30 µM) for 6 h (**a**) and 24 h (**b**) was assessed by means of cell counting (n = 6, each). Viability is expressed in % of the solvent control (EtOH; ctrl). **B**) Presence of CBD (30 µM; green) strongly increased the resting membrane potential of KGN cells whereas the solvent control EtOH (EtOH ctrl.; gray) had no impact, as demonstrated by changes in emission of the voltage sensitive fluorescence dye DiBAC4(3) (total change in fluorescence intensity during presence of 30 µM CBD 181.1 ± 8.2%). Relative fluorescence intensity over time (normalized to t_0_) of 36 examined KGN cells, with representative live cell images displayed as pseudo-color images (black-purple – relatively hyperpolarized; yellow-white – relatively depolarized) at the indicated time points (**c**-**f**). Scale bar 50 µm. **C**) Primary human granulosa cells (hGCs; boxed in cyan) and KGN cells (boxed in pink) were co-cultured alongside and treated with either 7 µM CBD (**c**) or the solvent control EtOH (**d**) for 24 h. Live cell imaging revealed induction of cell death exclusively in KGN cells. Representative micrographs with both cell types within the optical window at the start (top, t = 0 h) and the end of CBD treatment (bottom, t = 24 h). Scale bar 200 µm.

**
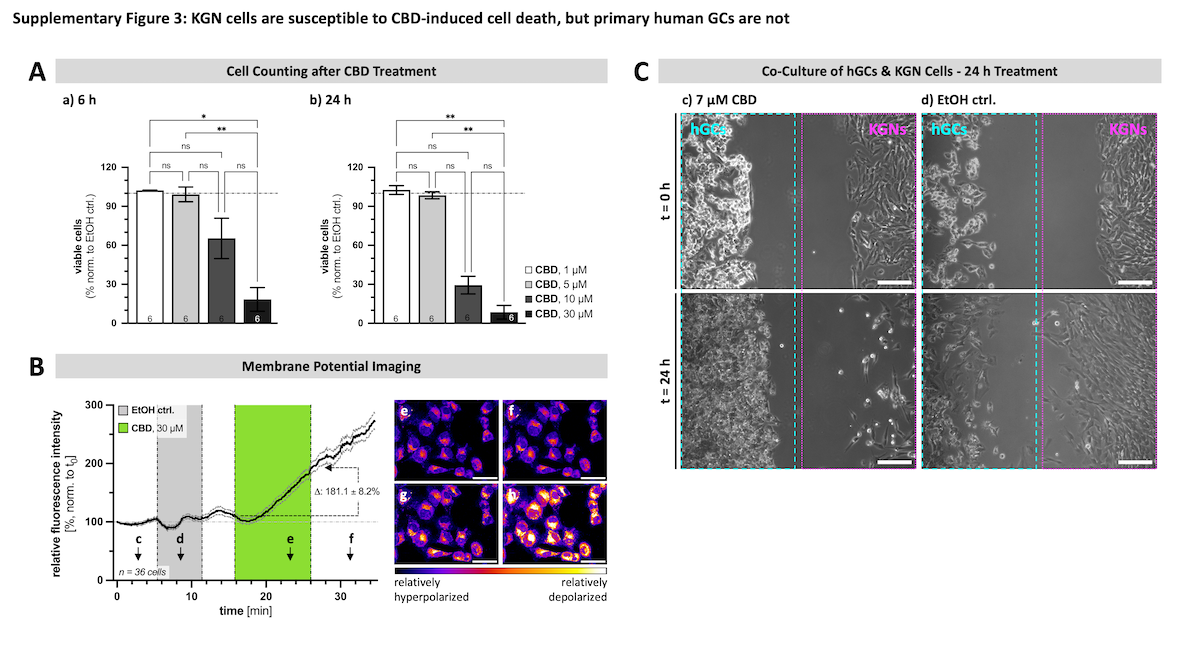
**

**Supplementary Figure 7: Absence of TRPV2 makes KGN cell less vulnerable to CBD-induced cell death and contribution of VDAC1 & mPTP to this action. A**) Representative micrographs of WT (top) and TRPV2-deficient clones #4 (middle) and #13 (bottom) captured at the end of a 6 h treatment with either 11 µM CBD (**a**) or the solvent control EtOH (**b**), revealing the appearance of cell death signs (nuclear condensation, intracellular vacuolization) only in WT cells. Scale bar 100 µm. **B**) Expression of VDAC1 in GCTs (**c**) and KGN cells (**d**) demonstrated by means of immunohistochemistry and Western Blotting, respectively. Cropped blot image shown original gel images are provided in **Supplementary Figure 9C.**) Representative micrographs from WT (**e**) and TRPV2-deficient clones #4 (**f**) and #13 (**g**) captured after 24 h treatment with the solvent control EtOH, corresponding to Figure 6B. **D**) Contribution of the mPTP to CBD-induced cell death was assessed by co-application of CysA (7 µM) and CBD (7 µM), followed by determination of cell viability by means of cell counting. Presence of CysA revealed to have a significantly protective effect for all three cell types and leveled any difference between those (n = 11-12, each).


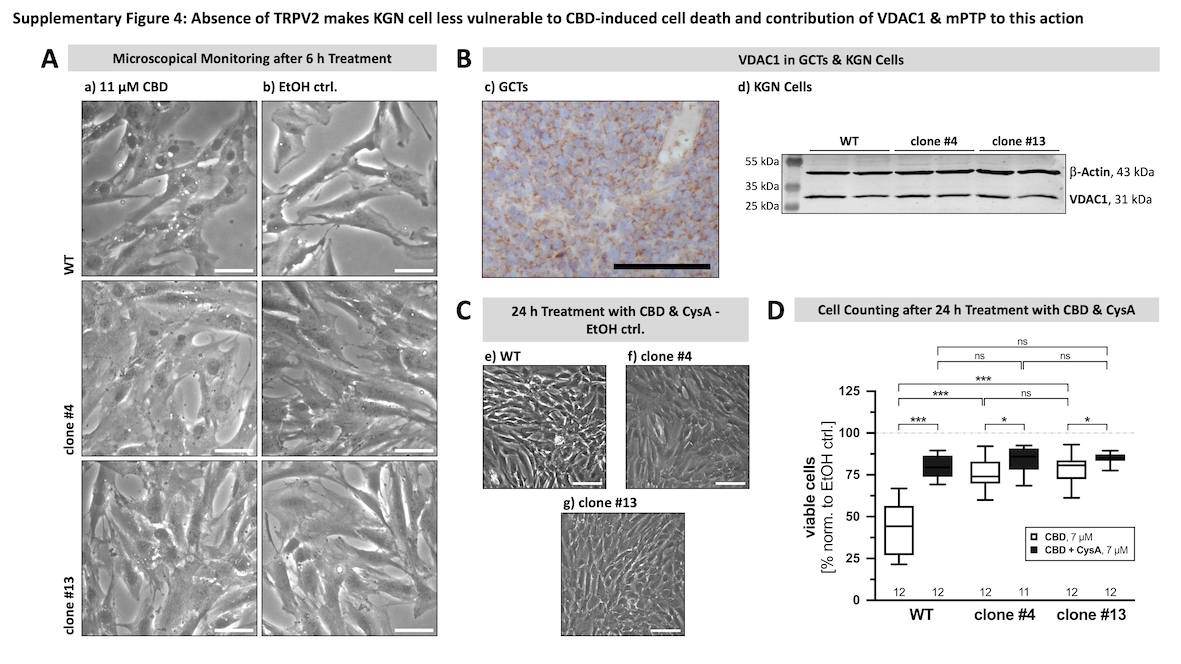


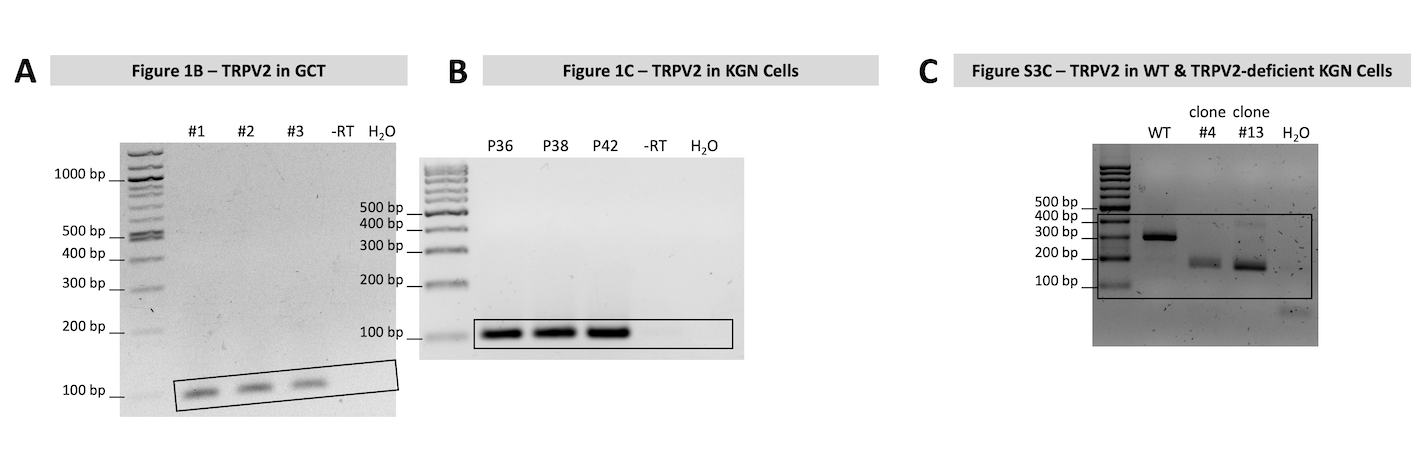
**Supplementary Figure 8: Original images of agarose gels. A**) Original agarose gel image with *TRPV2* PCR product bands sized 100 bp as obtained from GCTs. Three independent GCT samples were used. **B**) Original agarose gel image with *TRPV2* PCR product bands sized 100 bp as obtained from KGN RNA samples derived from three different passages (P36, P38 and P42). **C**) Original agarose gel image with *TRPV2* product bands sized ~290 bp in WT and ~170 bp in TRPV2-deficient clones #4 and #13, respectively. Omission of the reverse transcriptase during cDNA synthesis (-RT) or water instead of cDNA within the PCR reaction (H_2_O) served as negative controls. Any bands shown in the manuscript are boxed in black and the corresponding figure is stated in the header.


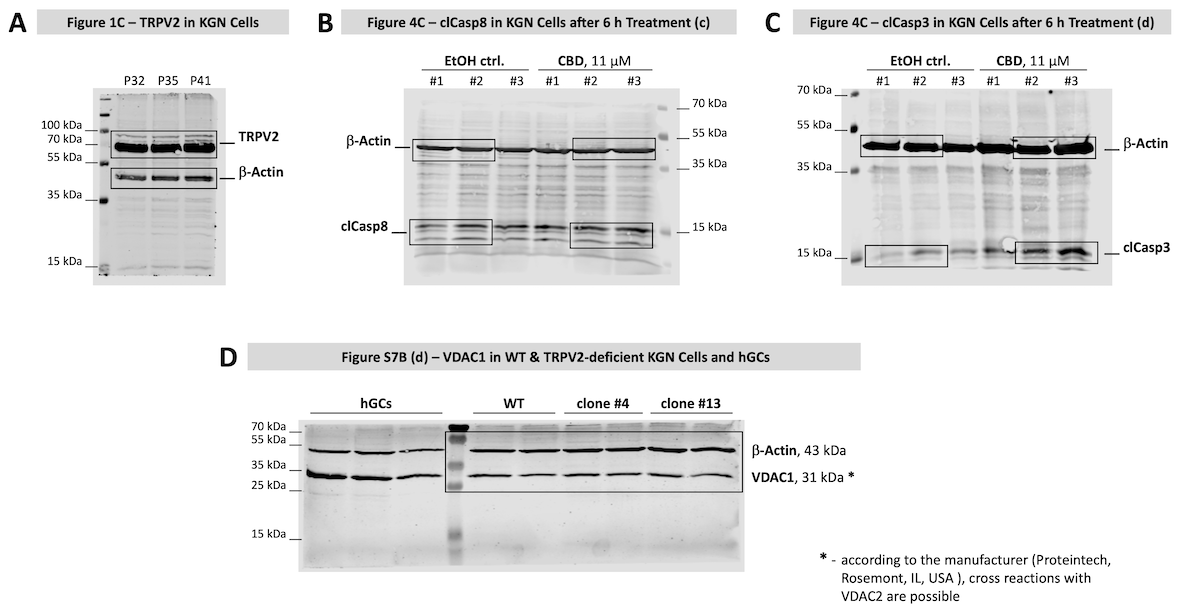
**Supplementary Figure 9 Original images of Western Blot membranes. A**) Original Western Blot membrane images from samples of different passages (P32, P35 and P41) derived from untreated WT KGN cells with TRPV2 bands. **B**) Original Western Blot membrane images with samples of WT KGN cells treated with solvent control EtOH or 11 µM CBD for 6 h (n = 3 replicates, each) with initiator clCasp8. **C**) Original Western Blot membrane images with samples of WT KGN cells treated with solvent control EtOH or 11 µM CBD for 6 h (n = 3 replicates, each) with effector clCasp3. **D**) Original Western Blot membrane images with samples of different passages (P32, P35 and P41) of untreated WT KGN cells and primary hGCs with a VDAC1* antibody. Detection of β-Actin served as internal loading control. Any bands shown in the manuscript are boxed in black and the corresponding figure is stated in the header.

**Supplementary Information**

**Mass Spectrometry of IP Samples**

For digestion, 500 ng modified porcine trypsin (Promega) in 100 µl 1 M urea/50 mM NH_4_HCO_3_ was added to the beads and shaken overnight at 37°C. The supernatant was collected and the peptides were extracted from the beads twice with 50 mM NH_4_HCO_3_. Cysteine residues were reduced with 1 mM 1,4-dithiothreitol for 10 min at 25°C, and alkylated for 30 min with iodoacetamide at 1.35 mM. To stop trypsinization, trifluoroacetic acid was added to give a final concentration of 1.25%, and samples were dried in a vacuum centrifuge concentrator. Liquid chromatography-tandem mass spectrometry (LC-MS/MS) analysis was performed with an UltiMate 3000 nano-LC system online coupled to a Q Exactive HF-X Orbitrap mass spectrometer (Thermo Fisher Scientific). LC-separation was conducted at 250 nl/min on an EASY-spray column (Pepmap™ RSLC, 75 μm × 50 cm, C18, 2 μm, Thermo Fisher Scientific). Solvent A comprised 0.1% FA in water. A two-step gradient from 3% to 25% B (0.1% FA in acetonitrile) over 30 min, followed by a ramp to 40% B in 5 min, was applied. Data were collected in data-dependent acquisition mode with a top 15 method. Raw files were processed using MaxQuant (1.6.11.0) with label-free quantification enabled and the Homo Sapiens subset of the Swiss-Prot database.

**Proteome Analysis of Whole Cell Lysates**

For mass spectrometry analysis of whole cell lysates, 1 x 10^6^ cells were lysed by ultrasonication for 15 min in 8 M urea/0.5 M NH_4_HCO_3_, and homogenized through QIAshredder devices (Qiagen). Protein contents were quantified using the Pierce 660 nm Protein Assay (Thermo Fisher Scientific). Disulfide bonds were reduced at 5 mM dithiothreitol for 30 min at 56 °C and alkylated at 15 mM iodoacetamide for 30 min at room temperature. Excess iodoacetamide was quenched with 1 mM dithiothreitol. Two step digestion was performed with Lys-C (FUJIFILM Wako Chemicals Europe GmbH, Neuss, Germany) for 4 h (1:100 enzyme to protein ratio) and then with modified porcine trypsin (Promega) for 16 h at 37°C (1:50 enzyme to protein ratio). Prior to trypsinization, samples were diluted with 50 mM NH_4_HCO_3_ to obtain 1 M urea. LC-MS/MS was performed with the same analytical setup as for the IP samples. The chromatography method included an 80-min gradient of 5–20% of solvent B followed by a 9 min increase to 40% and a 9 min step with 85% solvent B. MS acquisition was performed with a data independent acquisition method with 50 × 12 m/z-wide staggered windows (400–1000 m/z, 15,000 resolution, NCE 27). The main output file from DIA-NN, containing precursor-level data, was subsequently analyzed with the pepquantify R-package (https://github.com/bshashikadze/ pepquantify). This involved filtering for a 1% false-discovery rate as well as removal of potential contaminants, non-proteotypic peptides, and peptides with low signal quality. Proteins were filtered for at least 70% valid quantitative values in all samples. Data imputation of missing values was carried out using Perseus (v2.0.10.0). Significantly altered proteins were detected using a two-sample Student´s t-test with a significance cut-off (s0 = 0.1, FDR < 0.05).
